# Prognostic Factors for Cancer-Specific Survival and Disease-Free Interval of Dogs with Mammary Carcinomas

**DOI:** 10.1155/2023/6890707

**Published:** 2023-08-04

**Authors:** Elaine da Silva Soares, Fabrício Luciani Valente, Carolina Camargos Rocha, Carlos Eduardo Real Pereira, Thaís Barroso Sarandy, Fabiano Luiz Dulce de Oliveira, Sabrina Loise de Morais Calado, Andréa Pacheco Batista Borges

**Affiliations:** ^1^Department of Veterinary, Federal University of Viçosa (UFV), Viçosa (MG), Brazil; ^2^Faculty of Veterinary Medicine of the Rural Federal University of Rio de Janeiro (UFRRJ), Rio de Janeiro (RJ), Brazil; ^3^Department of Ecology and Conservation, Federal University of Paraná (UFPR), Curitiba (PR), Brazil

## Abstract

Canine mammary tumors (CMTs) are the most diagnosed neoplasms in dogs; however, there are few studies analyzing the influence of epidemiological, clinicopathological, and histopathological data on cancer-specific survival (CSS), disease-free interval (DFI), and overall survival (OS) in a large cohort. To contribute to the understanding of the biological behavior of this neoplasm, 385 cases were analyzed, 89% malignant, 4% benign, and 7% non-neoplastic lesions. Among the dogs diagnosed with malignant neoplasms, 86% had early clinical stages (I–III), while 14% had regional or distant metastasis at the time of diagnosis. Carcinoma in a mixed tumor was the most frequent histological type with 44% of the cases and had the best prognosis. Analyzed factors such as the presence of pseudocyesis, previous history of the disease, advanced clinical stage (IV-V), and presence of ulceration obtained significant results for CSS, DFI, and OS through univariate analysis and had a negative impact on the survival of the patients. Multivariate analysis showed that histological grading and age proved to be the best independent parameters for the prognostic evaluation of CSS and DFI in this study. These factors were also significant in the overall survival analysis. Therefore, these parameters should be considered valuable risk and prognostic factors for CMTs.

## 1. Introduction

Canine mammary tumors (CMTs) are the most common neoplasms in intact dogs and represent a serious problem in veterinary practice worldwide [1], with malignant tumors responsible for 50% to 70% of CMTs [1–4].

The definitive diagnosis of CMTs is performed through histopathological examination [5, 6]. Late diagnosis makes treatment difficult and reduces the survival of affected animals [7, 8]. The primary treatment to control CMTs is surgery [9–12], which aims to remove the tumor(s) with free margins and prevent the development of new tumors [1]. However, adjuvant therapies can be instituted after surgical treatment [13].

Knowledge of prognostic factors is important to determine therapeutic programs for cancer patients, as it allows the application of different therapeutic modalities in an appropriate and individualized way [14, 15] which can determine the success of the treatment and maintain the quality of life of cured patients [16]. Thus, the objective of the present study was to analyze risk factors and prognostic criteria through univariate and multivariate analyses of epidemiological data, clinicopathological and histopathological characteristics, and CSS and DFI of female dogs diagnosed with mammary tumors (MTs), to better understand the behavior of this neoplasm and assist in decision making.

## 2. Methods

### 2.1. Data Collection

Epidemiological and clinicopathological information of 385 female dogs submitted to mastectomy was obtained by consulting the medical records of the Veterinary Hospital of the Federal University of Viçosa (HVT-UFV), Minas Gerais/Brazil, from January 2012 to December 2020. The Ethics Committee for the Use of Animals of the Federal University of Viçosa (CEUA/UFV) approved this study and registered it under protocol number 18/2020.

The clinical data collected were as follows: age at diagnosis, body weight, breed, food type, body condition score (BCS), according to Baldwin et al. [17] on cachectic (1), thin (2), normal (3), fat (4), and obese (5), sterilization status, occurrence of pseudocyesis, hormone administration, and history of previous disease (mammary neoplasm, pyometra, or TVT (transmissible venereal tumor)). The clinicopathological variables analyzed were as follows: tumor location (thoracic, abdominal, and inguinal mammary glands), number of tumors (single or multiple), tumor size (T1: tumors <3 cm; T2: tumors between 3 and 5 cm; and T3: tumors >5 cm), presence of ulceration, regional lymph node status, and presence of distant metastasis.

Clinical staging was performed according to the TNM system (tumor size (T), lymph node involvement (N), and distant metastasis (M)) for CMTs and categorized into the following: stage I (T1N0M0, tumors <3 cm), stage II (T2N0M0, tumors between 3 and 5 cm), stage III (T3N0M0, tumors >5 cm), stage IV (any T-N1M0), and stage V (any T, any N0-1M1) [18, 19]. Finally, the animals were classified into early (I–III) or advanced (IV-V) clinical stages.

The staging and search for metastasis in distant organs were performed by chest radiography in the ventrodorsal and right and left laterolateral views, in addition to abdominal ultrasound evaluation, and routine tests such as blood cell count and biochemical profile were performed at the time of diagnosis of the mammary neoplasm. The treatment performed on the animals was exclusively surgical resection. The surgical procedures were classified as suggested by Fossum [20], as lumpectomy (removal of only the tumor), regional mastectomy (removal of the affected glands and the ones that shared lymphatic drainage, as well as the lymph node associated with the tumor), or radical mastectomy (unilateral removal of all the mammary chain and associated lymph nodes). The size, location, and number of tumors as well as the clinical status of regional lymph nodes determined the extension of resection.

### 2.2. Histological Processing and Classification of the Samples

The collected samples were fixed in a 10% buffered formalin solution for 48 hours, dehydrated in increasing solutions of ethyl alcohol, cleared in xylene, and embedded in paraffin. Histological sections (3 *μ*m) were obtained, and these sections were stained using the hematoxylin/eosin technique.

Tumors were classified according to the consensus for the diagnosis, prognosis, and treatment of CMTs [9, 12, 21]. In addition, the tumors were evaluated according to the Nottingham System classification [22]. The lesion with the worst histological classification and consequent worse prognosis was selected in animals with multiple tumors.

### 2.3. Animal Follow-Up

After surgery, patients were followed up every six months for a minimum period of 12 months, and the animal's owners were contacted by telephone in cases of loss of clinical follow-up. Local progression was categorized as recurrence close to the previous resection site. DFI was defined as the interval between surgery and the development of recurrences and/or nodal and distant metastasis. CSS was defined as the period between surgical excision of the tumor and disease-related death. OS was defined as the period from surgical removal of the tumor until the patient's death from any cause. Animals that were lost to follow-up were not considered for survival analyses.

### 2.4. Statistical Analysis

Comparisons of categorical variables were performed using the chi-square method. Parametric data were submitted for analysis of variance and Tukey's test, while non-parametric data were submitted to Kruskal–Wallis and Dunn's test, considering the significance of *P* < 0.05 in Prism 5.0 software (GraphPad Inc., CA, USA).

For data analysis, the Statistical Analysis System (SAS OnDemand) was used. The overall survival time was analyzed by survival analysis (Lifetest Procedure), the comparison among the strata was performed by log-rank and Wilcoxon tests, and the significance level adopted was *P* < 0.05.

Explanatory variables were used to predict the probability of survival for at least 1 year by multivariate logistic regression (Logistic Procedure), backward selection was used, and only variables that were significant at *P* < 0.10 were kept in the final model.

## 3. Results

### 3.1. Epidemiological and Clinicopathological Characteristics of Dogs Diagnosed with Benign and Malignant Neoplasms

Among the 385 cases analyzed, 7.01% (27/385) corresponded to non-neoplastic lesions, 4.16% (16/385) to benign neoplasms, and 88.83% (342/385) to malignant neoplasms. The median age and weight were 9.55 ± 2.91 years and 11.69 ± 9.97 kg, respectively, with overweight (BCS: 4) and obesity (BCS: 5) observed in 30.73% (110/358) of the cases, with a significant association between the body weight of animals with benign neoplasms and malignant neoplasms (*P*=0.034). Purebred animals represented 67.04% (240/358) of the cases, with Poodle (90/240), Pinscher (49/240), Yorkshire Terrier (15/240), German Shepherd (12/240), and Dachshund (12/240) being the most affected, while 32.96% (118/358) were considered mixed-breed (Table 1).

About 85.2% (305/358) were intact females, 14.8% (53/358) were spayed, 12.57% (45/358) had a history of pseudocyesis, and 17.6% (63/358) received unknown hormonal contraceptive agents. 46.37% (166/358) of the animals received commercial diets, while 31.28% (112/358) were fed a mixture of commercial dog food and homemade food. The occurrence of previous mammary neoplasms was reported in 18.16% (65/358) of the cases (Table 1).

Radical mastectomy was used in 47.21% (169/358) of cases and regional mastectomy in 37.71% (135/358). Concomitant to these procedures, ovariohysterectomy (OHE) was performed in 31.56% (113/358) of cases (Table 1). Single lesions corresponded to 24.02% (86/358) and multiple lesions represented 75.98% (272/358) of the cases, with 66.76% (239/358) located in more than one mammary gland, 15.92% (57/358) in the abdominal, and 13.13% (47/358) in the inguinal glands (Table 2).

Tumor size was correlated with the type of neoplasm, as most benign neoplasms (87.5%) and half of the malignant neoplasms (51.75%) were smaller than 3 cm in diameter (*P*=0.014). Ulceration, distant metastasis, and recurrence were observed, respectively, in 20.47% (70/358), 5.59% (20/358), and 17.88% (64/288) of malignant MTs. Clinicopathological information for the studied neoplasms is detailed in Table 2.

Among the non-neoplastic epithelial lesions, 51.85% (14/27) corresponded to lobular hyperplasia and 37.04% (10/27) to adenosis. Benign mixed tumors corresponded to 75% (12/16) of benign neoplasms, and among malignant neoplasms, carcinomas in a mixed tumor (169/342, 49.42%), tubular carcinomas (56/342, 16.37%), papillary carcinomas (34/342, 9.94%), and solid carcinomas (25/342, 7.31%) were the most frequent histological types. Among carcinomas of special types, micropapillary carcinomas represented 17/25 cases. Myoepithelial neoplasms represented 3.51% (12/342) of the cases, and carcinosarcoma was diagnosed in 2.92% (10/342) of the cases, being the most frequent among sarcomas (Supplementary Table 1).

### 3.2. Univariate Survival Analysis

#### 3.2.1. Cancer-Specific Survival (CSS) and Disease-Free Interval (DFI)

Animals with non-neoplastic lesions (*n* = 27) and benign neoplasms (*n* = 16) were excluded from survival analyses. The loss of clinical follow-up and contact with the animal owners occurred in 18.71% (64/342) of the dogs with malignant MTs. Among dogs with malignant MTs, the following were found after complete follow-up: (1) 27.34% (76/278) were alive without recurrence or metastasis, (2) 8.63% (24/278) were alive but with disease progression, (3) 26.98% (75/278) died due to mammary cancer, and (4) 37.05% (103/278) died of unrelated or unknown causes). As a result, 99 dogs (alive with recurrence and dead from the specific disease) were included in the CSS and DFI analyses, and for OS, 202 dogs were included.

A significant association was observed between age, CSS, and DFI. Younger animals (≤9.0 years) had higher median of CSS and DFI (1.460 days, confidence interval 95% IC 95% 730–1.825) and the older ones (>9.0 years) had the lowest (365 days, IC 95% 365–730, *P* = 0.001; *P* = 0.001, respectively, Supplementary Figures 1A and 1B; Supplementary Table 2). CSS and DFI did not significantly differ according to previous exposure to unknown hormonal contraceptives. CSS probabilities were not significantly different in animals with or without a history of pseudocyesis; however, animals with a history of pseudocyesis showed an increased DFI in comparison to those never experiencing pseudocyesis (*P* = 0.034, IC 95% 60-, Supplementary Figure 1C; Supplementary Table 2).

History of previous disease significantly influenced CSS, but not DFI. Dogs with a history of mammary neoplasm had a lower median of CSS (547 days, IC 95% 210–730) and animals without a history of previous disease had a higher median of CSS (730 days, IC 95% 365–1.095, *P*=0.021, Supplementary Figure 1D; Supplementary Table 2).

The type of food consumed by dogs with malignant carcinomas, tumor size, the presence or absence of distant metastases at time of the diagnosis, and the performance of OHE before or at the time of mastectomy were not significant for CSS and DFI.

Dogs in the initial clinical stage (I–III) had CSS and DFI (median of 730 days, IC 95% 730–1.095, *P*=0.006; *P*=0.013, Supplementary Figures 1E and 1F; Supplementary Table 2) significantly higher than those diagnosed at an advanced clinical stage (IV-V) (median of 365 days, IC 95% 90–730). Dogs diagnosed with carcinoma in a mixed tumor had higher median of CSS and DFI (1033 and 1095 days) than dogs with other types of malignant MTs.

The histological grading performed in 324 cases of malignant neoplasms resulted in the following: 36.73% (119/324) classified as grade I, 45.06% (146/324) as grade II, and 18.21% (59/324) as grade III, who had the lowest median of CSS and DFI (365 days, IC 95% 90–730; and 180 days, IC 95% 60–365, respectively). Histological grades I and II had significantly the highest median of CSS and DFI (*P* = 0.001; *P* = 0.002; Supplementary Figures 1G and 1H; Supplementary Table 2). Animals with ulcerated tumors at the time of diagnosis of the disease had a median CSS and DFI of 365 days (IC 95% 150–365), while in the absence of tumor ulceration, the median CSS and DFI was 730 days (IC 95% 730–1.095) (*P* = 0.003; *P* = 0.001; Supplementary Figures 1I and 1J; Supplementary Table 2). The type of procedure (radical or conservative surgery) performed did not demonstrate a significant prognostic value for CSS and DFI. CSS and DFI information for the malignant neoplasms studied is detailed in Table 3.

#### 3.2.2. Overall Survival (OS)

A significant association was observed between age and OS. Younger animals (≤9.0 years) had higher median of OS (1.095 days, IC 95% 730–1.825) and the older ones (>9.0 years) had the lowest (365 days, IC 95% 180–730, *P* ≤ 0.001, Supplementary Figure 2A). Animals with a history of pseudocyesis had a higher OS compared to those that never had pseudocyesis (*P*=0.039, IC 95% 365–1.825, Supplementary Figure 2B).

Dogs in the early clinical stage (I–III) had a significantly longer OS (median: 730 days, IC 95% 730–1.095) than those enrolled in an advanced clinical stage (IV-V) (median: 365 days; IC 95% 180–730, Supplementary Figure 2C). Histological grades I and II had significantly the highest median OS and grade III had the lowest median OS (365 days; IC 95% 210–730) (*P* ≤ 0.001; Supplementary Figure 2D). Animals with ulcerated tumors at the time of disease diagnosis had a median OS of 730 days (IC 95% 365–730), while in the absence of ulceration, the median OS was 730 days (IC 95% 730–1.095) (*P*=0.002; Supplementary Figure 2E).

OS was not influenced by hormone administration, tumor size, ovariohysterectomy, histological classification of tumors, and distant metastasis. OS information for the malignant neoplasms studied is detailed in Table 4.

### 3.3. Multivariate Survival Analysis

**Figure 1:**
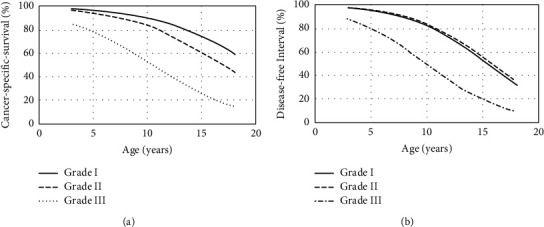
(a) Probability of CSS for at least 1 year in dogs affected by mammary tumor according to age and histological grade. (b) Probability of DFI for at least 1 year in dogs affected by mammary tumor according to age and histological grade.

The multivariate Cox regression model included the explanatory variables BCS (score 1–5), histological grade (score I–III), TNM-based clinical stage (initial or advanced), tumor size (score 1–3), pseudocyesis (present or absent), OHE (yes or no), distant metastasis (yes or no), ulceration (present or absent), hormone administration (yes or no), age (years), weight (kg), and number of mammary lesions (*n*). The model was able to explain significantly the variability seen in the population (*P* < 0.10). Only the histological grade and age variables remained as independent prognostic factors in the final model for CSS and DFI (Table 5; Figure 1).

In CSS, the odds ratio for age was 0.794. Each increase in age by 1 year multiplies the risk of event occurrence (cancer-related death) by 0.794, and thus each decrease in patient age by 1 year enhances the cancer-specific survival probabilities by 1.259 times. The odds ratio for the histological grade is given in Table 6.

In DFI, the odds ratio for age was 0.755. Each increase in age by 1 year multiplies the risk of event occurrence (cancer-related death) by 0.755, and thus each decrease in patient age by 1 year enhances the disease-free interval probabilities by 1.325 times. The odds ratio for histological grade is given in Table 6.

## 4. Discussion

Epidemiological studies and survival with multivariate analysis are scarce but important to elucidate the biological behavior of CMTs. They might be performed using veterinary records that allow the analysis of significant amounts of data, defining the prognostic and predictive factors, the characterization of neoplasms, and the observation of tumor progression, which are important for the definition of CSS and DFI and more appropriate therapy. Thus, the present study is of particular interest due to the cutoff of CMTs felt so far, making it a rare report on disease-free interval, specific survival, and overall survival of CMTs, as most previous studies have focused on the disease-free survival and overall survival only [23–30].

Age is considered a determining factor for the occurrence of MTs, more frequent between 9 and 11 years old [2, 19, 31–33], with a higher occurrence of malignant neoplasms in older animals [15] and benign neoplasms in young animals [4, 32]. In this study, a behavior similar to that described was observed.

Breed is also considered a risk factor for this disease as a result of the existence of a genetic predisposition [2, 34, 35]. In spite of this, in our study, we did not observe differences in CSS, DFI, and OS between mixed-breed and purebred animals with MTs, but as expected, purebred animals were more frequent [1, 4, 6].

The majority of dogs in this study were not spayed, which could be a risk factor for CSS, DFI, and OS since OHE in early life significantly reduces the risk of developing the CMTs [2, 34, 36, 37]. However, the performance or not of OHE in animals with MTs was not significant for CSS, DFI, and OS, as observed by Yamagami et al. [23] and Kristiansen et al. [38]. In addition, it was observed that the use of unknown hormonal contraceptive agents did not significantly influence CSS, DFI, and OS, different from what was expected, since the prolonged use of contraceptives stimulates the synthesis of growth hormone in the mammary gland [39].

Pseudocyesis is implicated in the pathogenesis of CMTs [40–42] and, in this study, proved to be a prognostic factor for DFI and OS. It is still not proven that dogs with a history of malignant MTs are at greater risk of developing new mammary neoplasms [43, 44]. However, it was observed that the history of MTs proved to be a prognostic factor for CSS.

Tumors occur more frequently in the caudal abdominal and inguinal mammary glands [45–47], probably because in these glands, there is a greater amount of mammary parenchyma and a greater proliferative response to the action of hormones [48], which corroborates the results of this study.

Tumor size is considered an independent prognostic factor, and T1 tumors are associated with a better prognosis [12, 15, 47]. T1 and T2 lesions occurred mainly in carcinoma in a mixed tumor and tubular carcinomas. Animals with nodal and distant metastasis (advanced clinical stage (IV-V)) had lower CSS compared to the initial stage (I–III) of the disease, in agreement with the findings of Nguyen et al. [15]. Animals with an advanced clinical stage (IV-V) should undergo combined therapy with chemotherapy adjuvant to surgery in an attempt to increase OS [12] and CSS.

In the present study, carcinoma in a mixed tumor was the most frequent histological type, corroborating the findings of other authors [8, 39, 49] and presenting a better prognosis and greater CSS, DFI, and OS. The histological type of the tumor must be taken into account, since histological variations confer differences in prognosis and treatment [21].

Most MTs in this study were associated with a low histological grade (I-II), resulting in better CSS, DFI, and OS. Tumors with the histological grade III showed a significant reduction in CSS, DFI, and OS, as observed by Peña et al. [29] and Nunes et al. [47] in dogs with undifferentiated carcinomas (grade III) that had a worse prognosis than dogs with grade I and II carcinomas. Supporting these data, the application of multivariate logistic regression selected histological grading as an important parameter to assess the clinical outcome. Histological grade was an independent and highly significant prognostic parameter for CSS, DFI, and OS.

Ulceration indicates a worse prognosis [9]. Ulceration can be caused by invasive tumor growth or trauma, ischemia, or skin infection, which are characteristics not necessarily associated with aggressive biological behavior [50]. In this study, the presence of tumor ulceration reduced the CSS of the affected animals by 50% and was mainly observed in patients with tumor size 3 and advanced clinical stage (IV-V). Thus, this clinical pathological feature can also be proposed as a prognostic factor for CMTs.

Surgery is the main treatment for MTs [12], except for those with inflammatory carcinomas [19, 51, 52]. CMTs are associated with high rates of morbidity and mortality [9, 15, 53]; in this study, a mortality rate of 21.63% was observed one year after the diagnosis of the mammary tumor.

We encountered multiple limitations throughout the process of making this research. For this retrospective study to have a larger number of cases analyzed and, therefore, more effective results, we looked over cases from the years 2012 to 2021, checking the hospital's records and manually selecting all of the female dogs that had a mastectomy. However, during this period, the Veterinary Hospital went through several renovations and changed the system, resulting in the loss of a big percentage of the information available from the previous years.

After that, we had to contact the animal owners of the animals we selected, as there was no follow-up described in the medical records. However, some telephone numbers were out of date or had not been provided and some guardians refused to participate in the study, so 60 animals were excluded due to failure to follow up.

Finally, we had all the information we wanted and selected the histological slides of each tumor to have a more recent and adequate histopathological classification, according to the consensus and literature suggested in the methodology [12]. Many slides disappeared and had to be redone after finding the paraffin blocks, but some materials were lost due to age or poor processing, excluding these animals from the study. After that, we had a total of 385 dogs with all the necessary factors to enter this retrospective study.

The only course of treatment used in these animals was the surgical removal of their respective tumors (radical or regional mastectomy or lumpectomy). The diagnostic method used consisted only of histological classification. Immunohistochemistry and other techniques are not widely used in our region due to the high cost and difficulty in acquiring antibodies. We recognize that the evaluation of biomarkers is extremely important to predict the biological behavior of cancer and to have a better prognosis for each case, but unfortunately, it is not possible to have it in the daily routine of the UFV Veterinary Hospital, which aims to make veterinary medicine accessible to the local population in need while teaching new professionals.

## 5. Conclusion

Advanced age and histological grading are important factors for the disease, both directly influencing the CSS, DFI, and OS of dogs with malignant neoplasm. Older animals had drastically reduced CSS and DFI compared to younger animals, and animals with grade I and II tumors had longer survival intervals than those with grade III. Female dogs with a history of previous mammary neoplasm had a lower median of CSS. Clinical staging significantly influenced survival, with dogs diagnosed in the early clinical stage having considerably higher CSS, DFI, and OS than those with advanced stage (IV-V). Furthermore, animals with ulcerated tumors had lower CSS, DFI, and OS compared to those with an absence of ulceration.

## Figures and Tables

**Table 1:** Epidemiological information and clinical staging of female dogs diagnosed with mammary gland tumors.

	Benign neoplasms (*n* = 16)	Malignant neoplasms (*n* = 342)	*P*
*Age*			
≤9.0 years	11 (68.75%)	163 (47.66%)	0.237
>9.0 years	5 (31.25%)	174 (50.88%)	
Undefined	0	5 (1.46%)	

*Breed*			
Mixed-breed	4 (25%)	114 (33.33%)	0.488
Purebred	12 (75%)	228 (66.67%)	

*Weight*			
≤10 kg	12 (75%)	207 (60.53%)	0.034
>10 kg	4 (25%)	135 (39.47%)	

*BCS*			
1	0	1 (0.29%)	0.402
2	0	16 (4.69%)	
3	8 (50%)	222 (65.1%)	
4	7 (43.75%)	79 (23.17%)	
5	1 (6.25%)	23 (6.74%)	

*Type of food*			
Homemade food	0	14 (4.09%)	0.379
Commercial diets	9 (56.25%)	157 (45.91%)	
Commercial diets + meat	1 (6.25%)	5 (1.46%)	
Commercial diets + homemade food	5 (31.25%)	107 (31.29%)	
Commercial diets + snack	1 (6.25%)	59 (17.25%)	

*Ovariohysterectomy (OHE)*			
No	13 (81.25%)	292 (85.38%)	0.649
Yes	3 (18.75%)	50 (14.62%)	

*Pseudocyesis*			
Absent	15 (93.75%)	298 (87.13%)	0.435
Present	1 (6.25%)	44 (12.87%)	

*Received unknown hormonal contraceptive agents*			
No	12 (75%)	283 (82.75%)	0.426
Yes	4 (25%)	59 (17.25%)	

*History of previous disease*			
Denied	10 (62.5%)	265 (77.49%)	0.075
Mammary neoplasm	4 (25%)	61 (17.84%)	
Pyometra	1 (6.25%)	14 (4.09%)	
TVT	1 (6.25%)	2 (0.58%)	

*Surgical technique*			
Lumpectomy	1 (6.25%)	21 (6.14%)	0.544
Regional mastectomy	6 (37.5%)	129 (37.72%)	
Radical mastectomy	6 (37.5%)	163 (47.66%)	
Combination of techniques	3 (18.75%)	29 (8.48%)	

*Concomitant OHE*			
No	11 (68.75%)	234 (68.42%)	0.978
Yes	5 (31.25%)	108 (31.58%)	

BCS, body condition score; TVT, transmissible venereal tumor. Similar group characteristics are verified by nonsignificant *P* < 0.05 using the chi-square test on categorized variables.

**Table 2:** Clinicopathological characteristics evaluated in female dogs diagnosed with mammary gland tumors.

	Benign neoplasms (*n* = 16)	Malignant neoplasms (*n* = 342)	*P*
*Ulceration*			
Absent	16 (100%)	272 (79.53%)	0.044
Present	0	70 (20.47%)	

*Tumor size*			
*T*1	14 (87.5%)	177 (51.57%)	0.014
*T*2	2 (12.5%)	69 (20.18%)	
*T*3	0	96 (28.07%)	

*Number of tumors*			
Single	2 (12.5%)	84 (24.56%)	0.270
Multiple	14 (87.5%)	258 (75.44%)	

*Location of the tumor*			
Thoracic	0	15 (4.39%)	0.338
Abdominal	1 (6.25%)	56 (16.37%)	
Inguinal	1 (6.25%)	46 (13.45%)	
Multicenter	14 (87.5%)	225 (65.79%)	

*Distant metastasis*			
No	15 (93.75%)	323 (94.44%)	0.906
Yes	1 (6.25%)	19 (5.56%)	

*Local recurrence*			
No	9 (90%)	215 (77.34%)	0.154
Yes	1 (10%)	63 (22.66%)	

*T*1, tumors <3 cm; *T*2, tumors between 3and 5 cm; *T*3, tumors >5 cm. Similar group characteristics are verified by nonsignificant *P* < 0.05 using the chi-square test on categorized variables.

**Table 3:** One-year corrected survival rates in 95 cases (4 failures) with available follow-up data^a^.

		Cancer-specific survival (*n* = 95)			Disease-free interval (*n* = 95)		
		*n*	Median survival (days)	Median 1-yearsurvival rate *n* (%)	*n*	Median survival (days)	Median 1-yearsurvival rate *n* (%)
Age	≤9.0 years	30	1.460	23 (76.67%)	30	1.460	23 (76.67%)
	>9.0 years	65	365	31 (47.69%)	65	365	31 (47.69%)
	*P*		**0.001**			**0.001**	

Breed	Mixed-breed	27	730	15 (55.56%)	27	365	13 (48.15%)
	Purebred	68	730	39 (57.35%)	68	730	35 (66.18%)
	*P*		0.453			0.234	

Type of food	Homemade food	2	380	0	2	197	0
	Commercial diets	43	730	24 (55.81%)	43	730	23 (53.49%)
	Commercial diets + meat	2	2.007	1 (50%)	2	2.007	1 (50%)
	Commercial diets + homemade food	35	365	17 (48.57%)	35	365	14 (40%)
	Commercial diets + snack	13	1.095	10 (76.92%)	13	1.095	9 (69.23%)
	*P*		0.117			0.082	

Ovariohysterectomy (OHE)	No	78	730	47 (60.26%)	78	730	42 (53.85%)
	Yes	17	365	7 (41.17%)	17	365	6 (35.29%)
	*P*		0.090			0.078	

Pseudocyesis	Absent	88	730	50 (56.82%)	88	365	43 (48.86%)
	Present	7	1.825	4 (57.14%)	7	1.825	4 (57.14%)
	*P*		0.182			**0.034**	

Hormone administration	No	81	730	45 (55.56%)	81	730	40 (49.38%)
	Yes	14	730	9 (64.29%)	14	730	8 (57.14%)
	*P*		0.714			0.636	

History of previous disease	Negative	71	730	42 (59.15%)	71	730	37 (52.11%)
	Positive	24	547	12 (50%)	24	365	11 (45.83%)
	*P*		**0.021**			0.230	

Clinical stage (TNM)	Initial (I–III)	74	730	45 (60.81%)	74	730	40 (54.05%)
	Advanced (IV-V)	21	365	9 (42.86%)	21	365	8 (38.1%)
	*P*		**0.006**			**0.013**	

Surgical technique	Radical mastectomy	48	730	27 (56.25%)	48	730	25 (52.08%)
	Other techniques	47	730	27 (57.45%)	47	365	23 (48.94%)
	*P*		0.782			0.937	

Tumor size	*T*1 (<2 cm)	49	730	27 (55.1%)	49	730	24 (48.98%)
	*T*2 (2-3 cm)	20	547	10 (50%)	20	365	9 (45%)
	*T*3 (>3 cm)	26	730	17 (65.38%)	26	730	15 (57.69%)
	*P*		0.668			0.698	

Histological type (*n* = 72)	Carcinoma in a mixed tumor	42	1.033	31 (73.81%)	42	1.095	27 (64.29%)
	Solid carcinoma	15	365	6 (40%)	15	365	6 (40%)
	Tubular carcinoma	11	730	6 (54.55%)	11	365	5 (45.45%)
	*P*		0.298			0.264	

Histological grade (*n* = 90)	Grade I	32	1.095	22 (68.75%)	32	1.095	21 (65.63%)
	Grade II	27	1.095	19 (70.37%)	27	1.095	16 (59.62%)
	Grade III	31	365	11 (35.48%)	31	180	9 (29.03%)
	*P*		**0.001**			**0.002**	

Distant metastasis	No	84	730	48 (58.54%)	84	730	42 (50%)
	Yes	11	730	6 (54.55%)	11	730	6 (54.55%)
	*P*		0.133			0.195	

Ulceration	Absent	67	730	43 (64.18%)	67	730	40 (59.7%)
	Present	28	365	11 (39.29%)	28	365	8 (28.57%)
	*P*		**0.003**			**0.001**	

^a^Survival values are percentages for cumulative survival determined by the log-rank test. Significance level adopted was *P* < 0.05. *P* values in bold indicate significance.

**Table 4:** One-year corrected survival rates in 202 cases with available follow-up data^a^.

		Overall survival (*n* = 202)		
		*n*	Median survival (days)	Median 1-year survival rate *n* (%)
Age	≤9.0 years	30	1.095	16 (53.33%)
	>9.0 years	65	365	18 (27.69%)
	*P*	**<0.001**		

Ovariohysterectomy (OHE)	No	173	730	68 (39.31%)
	Yes	29	730	11 (37.93%)
	*P*	0.355		

Pseudocyesis	Absent	184	730	70 (38.04%)
	Present	18	1.095	8 (44.44%)
	*P*	**0.039**		

Hormone administration	No	170	730	70 (41.18%)
	Yes	32	730	9 (28.13%)
	*P*	0.865		

Clinical stage (TNM)	Initial (I–III)	170	730	72 (42.35%)
	Advanced (IV–V)	32	365	15 (%)
	*P*	**0.031**		

Tumor size	*T*1 (<2 cm)	94	730	35 (37.23%)
	*T*2 (2-3 cm)	45	730	18 (40%)
	*T*3 (>3 cm)	63	730	26 (41.27%)
	*P*	0.749		

Histological type (*n* = 145)	Carcinoma in a mixed tumor	90	730	40 (44.44%)
	Solid carcinoma	19	365	8 (42.11%)
	Tubular carcinoma	36	730	12 (33.33%)
	*P*	0.205		

Histological grade (*n* = 186)	Grade I	66	1.095	16 (24.24%)
	Grade II	77	730	35 (45.45%)
	Grade III	43	365	20 (46.51%)
	*P*	**<0.001**		

Distant metastasis	No	186	730	75 (40.32%)
	Yes	16	547	4 (25%)
	*P*	0.143		

Ulceration	Absent	153	730	65 (42.48%)
	Present	49	730	14 (28.57%)
	*P*	**0.002**		

^a^Survival values are percentages for cumulative survival determined by the log-rank test. Significance level adopted was *P* < 0.05. *P* values in bold indicate significance.

**Table 5:** Analysis of maximum likelihood estimates of multivariate logistic regression to predict the probability of overall survival and disease-free interval for at least 1 year after surgery in dogs affected by mammary tumor.

Parameter	Estimate	Standard error
*Cancer-specific survival*		
Intercept	3.6329	1.0338
Grade (1)	0.9109	0.4777
Grade (2)	0.2941	0.4453
Grade (3)	—	—
Age	−0.2307	0.0947
*c* (area under ROC curve) = 0.816		

*Disease-free interval*		
Intercept	3.8524	1.0272
Grade (1)	0.4808	0.3946
Grade (2)	0.5794	0.4269
Grade (3)	—	—
Age	−0.2811	0.0956
*c* (area under ROC curve) = 0.823		

**Table 6:** Odds ratio for histological grade.

Histological grade	1	2	3
*Cancer-specific survival*			
1	—	1.853	8.297
2		—	4.478
3			—

*Disease-free interval*			
1	—	0.906	4.669
2		—	5.153
3			—

## Data Availability

The data used to support the findings of this study are available on request from the corresponding author.

## Supplementary Materials

Supplementary Materials Supplementary Figure 1: cancer-specific survival and disease-free interval Kaplan–Meier curve of female dogs with malignant mammary neoplasms. Malignant mammary neoplasms are classified according to (A) CSS age: ≤9 years (median: 1.460 days) and >9 years (median: 365 days); (B) DFI age: ≤9 years (median: 1.460 days) and >9 years (median: 365 days); (C) DFI pseudocyesis: present (median: 1.825 days) and absent (median: 730 days); (D) CSS disease history: negative (median: 730 days) and positive (median: 547 days); (E) CSS clinical staging: initial (median: 730 days) and advanced (365 days); (F) DFI clinical staging: initial (median: 730 days) and advanced (365 days); (G) CSS histological grade: I (median: 1.095 days), II (1.095 days), and III (365 days), respectively; (H) DFI histological grade: I (median: 1.095 days), II (1.095 days), and III (180 days), respectively; (I) CSS ulceration: present (median: 365 days) and absent (median: 730 days); (J) DFI ulceration: present (median: 365 days) and absent (median: 730 days). Supplementary Figure 2: overall survival Kaplan–Meier curve of female dogs with malignant mammary neoplasms. Malignant mammary neoplasms are classified according to (A) age: ≤9 years (median: 1.095 days) and >9 years (median: 365 days); (B) pseudocyesis: present (median: 1.095 days) and absent (median: 730 days); (C) clinical staging: initial (median: 730 days) and advanced (365 days); (D) histological grade: I (median: 1.095 days), II (730 days), and III (365 days), respectively; (E) ulceration: present (median: 730 days) and absent (median: 730 days). Supplementary Table 1: histopathological classification and frequency in % of 385 cases of mammary tumors diagnosed in female dogs treated at the UFV Veterinary Hospital, which were classified into non-neoplastic lesions (*n* = 27/7.01%), benign neoplasms (*n* = 16/4.16%), and malignant neoplasms (*n* = 342/88.83%). Supplementary Table 2: estimates of life survival and risk functions in 95 cases with available follow-up data: age ≤9.0 years (*n* = 30) and age >9.0 years (*n* = 65); history of previous disease: negative (*n* = 71) and positive (*n* = 24); clinical stage (TNM): initial (I–III) (*n* = 74) and advanced (IV-V) (*n* = 21); histological grade (*n* = 90): grade I (*n* = 32), grade II (*n* = 27), and grade III (*n* = 31); ulceration: absent (*n* = 67) and present (*n* = 28), for cancer-specific survival. Estimates of overall lifetime survival and risk functions in 95 cases with available follow-up data: age ≤9.0 years (*n* = 30) and age >9.0 years (*n* = 65); pseudocyesis: absent (*n* = 88) and present (*n* = 7); clinical stage (TNM): initial (I–III) (*n* = 74) and advanced (IV-V) (*n* = 21); histological grade (*n* = 90): grade I (*n* = 32), grade II (*n* = 27), and grade III (*n* = 31); ulceration: absent (*n* = 67) and present (*n* = 28), for disease-free interval.
