# Supplementary material for: Prognostic Factors for Cancer-Specific Survival and Disease-Free Interval of Dogs with Mammary Carcinomas
Source: Vet Med Int. 2023 Aug 4;2023:6890707. doi: 10.1155/2023/6890707 (PMC10421712; doi:10.1155/2023/6890707)
Supplement: Supplementary Materials — Supplementary Figure 1: cancer-specific survival and disease-free interval Kaplan–Meier curve of female dogs with malignant mammary neoplasms. Malignant mammary neoplasms are classified according to (A) CSS age: ≤9 years (median: 1.460 days) and >9 years (median: 365 days); (B) DFI age: ≤9 years (median: 1.460 days) and >9 years (median: 365 days); (C) DFI pseudocyesis: present (median: 1.825 days) and absent (median: 730 days); (D) CSS disease history: negative (median: 730 days) and positive (median: 547 days); (E) CSS clinical staging: initial (median: 730 days) and advanced (365 days); (F) DFI clinical staging: initial (median: 730 days) and advanced (365 days); (G) CSS histological grade: I (median: 1.095 days), II (1.095 days), and III (365 days), respectively; (H) DFI histological grade: I (median: 1.095 days), II (1.095 days), and III (180 days), respectively; (I) CSS ulceration: present (median: 365 days) and absent (median: 730 days); (J) DFI ulceration: present (median: 365 days) and absent (median: 730 days). Supplementary Figure 2: overall survival Kaplan–Meier curve of female dogs with malignant mammary neoplasms. Malignant mammary neoplasms are classified according to (A) age: ≤9 years (median: 1.095 days) and >9 years (median: 365 days); (B) pseudocyesis: present (median: 1.095 days) and absent (median: 730 days); (C) clinical staging: initial (median: 730 days) and advanced (365 days); (D) histological grade: I (median: 1.095 days), II (730 days), and III (365 days), respectively; (E) ulceration: present (median: 730 days) and absent (median: 730 days). Supplementary Table 1: histopathological classification and frequency in % of 385 cases of mammary tumors diagnosed in female dogs treated at the UFV Veterinary Hospital, which were classified into non-neoplastic lesions (n = 27/7.01%), benign neoplasms (n = 16/4.16%), and malignant neoplasms (n = 342/88.83%). Supplementary Table 2: estimates of life survival and risk functions in 95 cases with avail [file 6890707.f1.zip › 6890707.f1/Supplementary Table 1 (3).docx]

**Supplementary Table 1.** Histological classification and frequency of mammary tumors diagnosed in female dogs.

| Tumors Classification | Histological Classification | N=385 | % |
| --- | --- | --- | --- |
|  | 1.1 Adenosis | 10 | 2.60 |
| **1. Non-neoplastic lesions** | 1.2 Ductal ectasia | 2 | 0.52 |
| N = 27 (7.01%) | 1.3 Ductal hyperplasia | 1 | 0.26 |
|  | 1.4 lobular hyperplasia | 14 | 3.64 |
|  | 2.1 Adenoma | 3 | 0.78 |
| **2. Benign Neoplasms** | 2.2 Adenomyoepithelioma | 1 | 0.26 |
| N = 16 (4.16%) | 2.3 Benign mixed tumor | 12 | 3.12 |
|  | **3.1 Carcinomas** |  |  |
|  | 3.1.1 Carcinoma in situ | 1 | 0.26 |
|  | 3.1.2 Basaloid carcinoma | 1 | 0.26 |
|  | 3.1.3 Cribriform carcinoma | 2 | 0.52 |
|  | 3.1.4 Carcinoma in a mixed tumor | 169 | 43.90 |
|  | 3.1.5 Papillary carcinoma | 34 | 8.83 |
|  | 3.1.6 Tubular carcinoma | 56 | 14.55 |
|  | 3.1.7 Solid carcinoma | 25 | 6.49 |
| **3. Malignant Neoplasms** | **3.2 Special type Carcinomas** |  |  |
| N = 342 (88.83%) | 3.2.1 Pleomorphic lobular carcinoma | 4 | 1.04 |
|  | 3.2.2 Micropapillary carcinoma | 17 | 4.42 |
|  | 3.2.3 Mucinous carcinoma | 1 | 0.26 |
|  | 3.2.4 Lipid-rich carcinoma | 1 | 0.26 |
|  | 3.2.5 Secretory carcinoma | 1 | 0.26 |
|  | 3.2.6 Squamous cell carcinoma | 2 | 0.52 |
|  | **3.3 Myoepithelial Neoplasms** |  |  |
|  | 3.3.1 Malignant adenomyoepithelioma | 10 | 2.60 |
|  | 3.3.2 Malignant myoepithelioma | 2 | 0.52 |
|  | **3.4 Sarcomas** |  |  |
|  | 3.4.1 Carcinosarcoma | 10 | 2.60 |
|  | 3.4.2 Chondrosarcoma | 3 | 0.78 |
|  | 3.4.3 Hemangiosarcoma | 2 | 0.52 |
|  | 3.4.4 Osteosarcoma | 1 | 0.26 |
