# Supplementary material for: Prognostic Factors for Cancer-Specific Survival and Disease-Free Interval of Dogs with Mammary Carcinomas
Source: Vet Med Int. 2023 Aug 4;2023:6890707. doi: 10.1155/2023/6890707 (PMC10421712; doi:10.1155/2023/6890707)
Supplement: Supplementary Materials — Supplementary Figure 1: cancer-specific survival and disease-free interval Kaplan–Meier curve of female dogs with malignant mammary neoplasms. Malignant mammary neoplasms are classified according to (A) CSS age: ≤9 years (median: 1.460 days) and >9 years (median: 365 days); (B) DFI age: ≤9 years (median: 1.460 days) and >9 years (median: 365 days); (C) DFI pseudocyesis: present (median: 1.825 days) and absent (median: 730 days); (D) CSS disease history: negative (median: 730 days) and positive (median: 547 days); (E) CSS clinical staging: initial (median: 730 days) and advanced (365 days); (F) DFI clinical staging: initial (median: 730 days) and advanced (365 days); (G) CSS histological grade: I (median: 1.095 days), II (1.095 days), and III (365 days), respectively; (H) DFI histological grade: I (median: 1.095 days), II (1.095 days), and III (180 days), respectively; (I) CSS ulceration: present (median: 365 days) and absent (median: 730 days); (J) DFI ulceration: present (median: 365 days) and absent (median: 730 days). Supplementary Figure 2: overall survival Kaplan–Meier curve of female dogs with malignant mammary neoplasms. Malignant mammary neoplasms are classified according to (A) age: ≤9 years (median: 1.095 days) and >9 years (median: 365 days); (B) pseudocyesis: present (median: 1.095 days) and absent (median: 730 days); (C) clinical staging: initial (median: 730 days) and advanced (365 days); (D) histological grade: I (median: 1.095 days), II (730 days), and III (365 days), respectively; (E) ulceration: present (median: 730 days) and absent (median: 730 days). Supplementary Table 1: histopathological classification and frequency in % of 385 cases of mammary tumors diagnosed in female dogs treated at the UFV Veterinary Hospital, which were classified into non-neoplastic lesions (n = 27/7.01%), benign neoplasms (n = 16/4.16%), and malignant neoplasms (n = 342/88.83%). Supplementary Table 2: estimates of life survival and risk functions in 95 cases with avail [file 6890707.f1.zip › 6890707.f1/Supplementary Table 2 (1).docx]

**Supplementary Table 2.** Life Survival Estimates and hazard functions in 95 cases with available follow-up data.

|  | **Cancer-specifc survival** (*n*=95) | | | | | | | | | | |
| --- | --- | --- | --- | --- | --- | --- | --- | --- | --- | --- | --- |
|  | Interval | | Number  Failed | Number  Censored | Suvival | Failure | Survival  Standard  Error | Evaluated At the Midpoint of the Interval | | | |
|  | Lower | Upper |  |  |  |  |  | PDF | PDF  Standard  Error | Hazard | Hazard  Standard  Error |
| **Age ≤9.0 years** (*n = 30)* | 0 | 500 | 10 | 4 | 1.0000 | 0 | 0 | 0.000455 | 0.000126 | 0.000513 | 0.000161 |
|  | 500 | 1000 | 6 | 5 | 0.7727 | 0.2273 | 0.0632 | 0.000314 | 0.000117 | 0.000453 | 0.000184 |
|  | 1000 | 1500 | 8 | 7 | 0.6156 | 0.3844 | 0.0762 | 0.000563 | 0.000162 | 0.001185 | 0.0004 |
|  | 1500 | 2000 | 4 | 1 | 0.3342 | 0.6658 | 0.0842 | 0.000486 | 0.000176 | 0.002286 | 0.000938 |
|  | 2000 | 2500 | 1 | 0 | 0.0911 | 0.9089 | 0.0675 | 0.000182 | 0.000135 | 0.004 | 0 |
|  | 2500 |  | 0 | 0 | 0 | 1.0000 | 0 |  |  |  |  |
|  | | | | | | | | | | | |
| **Age ˃9.0 years** (*n = 65*) | 0 | 500 | 29 | 3 | 1.0000 | 0 | 0 | 0.000122 | 0.000142 | 0.001758 | 0.000293 |
|  | 500 | 1000 | 9 | 1 | 0.3895 | 0.6105 | 0.0708 | 0.000425 | 0.000123 | 0.0015 | 0.000464 |
|  | 1000 | 1500 | 4 | 3 | 0.1770 | 0.8230 | 0.0576 | 0.000258 | 0.000107 | 0.002286 | 0.000938 |
|  | 1500 |  | 0 | 0 | 0.0483 | 0.9517 | 0.0371 |  |  |  |  |
|  | | | | | | | | | | | |
| **History of previous disease – Negative**  (*n = 71*) | 0 | 500 | 29 | 5 | 1.0000 | 0 | 0 | 0.000800 | 0.000115 | 0.001 | 0.00018 |
|  | 500 | 1000 | 9 | 6 | 0.6000 | 0.4000 | 0.0575 | 0.000284 | 0.000087 | 0.000537 | 0.000177 |
|  | 1000 | 1500 | 11 | 9 | 0.4579 | 0.5421 | 0.0603 | 0.000469 | 0.000116 | 0.001375 | 0.000389 |
|  | 1500 | 2000 | 4 | 1 | 0.2236 | 0.7764 | 0.0575 | 0.000325 | 0.000119 | 0.002286 | 0.000938 |
|  | 2000 | 2500 | 1 | 0 | 0.0610 | 0.9390 | 0.0453 | 0.000122 | 0.000091 | 0.004 | 0 |
|  | 2500 |  | 0 | 0 | 0 | 1.0000 | 0 |  |  |  |  |
|  | | | | | | | | | | | |
| **History of previous disease – Positive**  (*n =24*) | 0 | 500 | 10 | 2 | 1.0000 | 0 | 0 | 0.00105 | 0.000229 | 0.001429 | 0.000422 |
|  | 500 | 1000 | 6 | 0 | 0.4737 | 0.5263 | 0.1145 | 0.000711 | 0.000225 | 0.0024 | 0.000784 |
|  | 1000 | 1500 | 1 | 1 | 0.1184 | 0.8816 | 0.0780 | 0.000158 | 0.000138 | 0.002 | 0.001732 |
|  | 1500 |  | 0 | 0 | 0.0395 | 0.9605 | 0.0525 |  |  |  |  |
|  | | | | | | | | | | | |
| **Clinical Stage** (TNM)  Initial (I–III)  (*n= 74*) | 0 | 500 | 27 | 7 | 1.0000 | 0 | 0 | 0.000766 | 0.000116 | 0.000947 | 0.000177 |
|  | 500 | 1000 | 9 | 5 | 0.6170 | 0.3830 | 0.0579 | 0.000296 | 0.000090 | 0.000545 | 0.00018 |
|  | 1000 | 1500 | 11 | 9 | 0.4689 | 0.5311 | 0.0615 | 0.000480 | 0.000119 | 0.001375 | 0.000389 |
|  | 1500 | 2000 | 4 | 1 | 0.2290 | 0.7710 | 0.0588 | 0.000333 | 0.000122 | 0.002286 | 0.000938 |
|  | 2000 | 2500 | 1 | 0 | 0.0625 | 0.9375 | 0.0464 | 0.000125 | 0.000093 | 0.004 | 0 |
|  | 2500 |  | 0 | 0 | 0 | 1.0000 | 0 |  |  |  |  |
|  | | | | | | | | | | | |
| **Clinical Stage** (TNM)  Advanced (IV–V)  (*n=21* ) | 0 | 500 | 12 | 0 | 1.0000 | 0 | 0 | 0.00114 | 0.000216 | 0.0016 | 0.000423 |
|  | 500 | 1000 | 6 | 1 | 0.4286 | 0.5714 | 0.1080 | 0.000605 | 0.000203 | 0.002182 | 0.000747 |
|  | 1000 | 1500 | 1 | 1 | 0.1261 | 0.8739 | 0.0741 | 0.000168 | 0.000139 | 0.002 | 0.001732 |
|  | 1500 |  | 0 | 0 | 0.0420 | 0.9580 | 0.0544 |  |  |  |  |
|  | | | | | | | | | | | |
| **Histological grade**  **(*n = 90*)**  Grade I  (*n= 32*) | 0 | 500 | 8 | 7 | 1.0000 | 0 | 0 | 0.000561 | 0.000168 | 0.000653 | 0.000228 |
|  | 500 | 1000 | 4 | 2 | 0.7193 | 0.2807 | 0.0842 | 0.000360 | 0.000161 | 0.000571 | 0.000283 |
|  | 1000 | 1500 | 4 | 6 | 0.5395 | 0.4605 | 0.1002 | 0.000539 | 0.000215 | 0.001333 | 0.000629 |
|  | 1500 | 2000 | 1 | 0 | 0.2697 | 0.7303 | 0.1077 | 0.000539 | 0.000215 | 0.004 | 0 |
|  | 2000 |  | 0 | 0 | 0 | 1.0000 | 0 |  |  |  |  |
|  | | | | | | | | | | | |
| **Histological grade**  **(*n = 90*)**  Grade II  (*n= 27*) | 0 | 500 | 8 | 0 | 1.0000 | 0 | 0 | 0.000593 | 0.000176 | 0.000696 | 0.000242 |
|  | 500 | 1000 | 4 | 4 | 0.7037 | 0.2963 | 0.0879 | 0.000331 | 0.000151 | 0.000533 | 0.000264 |
|  | 1000 | 1500 | 5 | 2 | 0.5381 | 0.4619 | 0.0988 | 0.000538 | 0.000197 | 0.001333 | 0.000562 |
|  | 1500 | 2000 | 3 | 1 | 0.2691 | 0.7309 | 0.0984 | 0.000461 | 0.000196 | 0.003 | 0.001146 |
|  | 2000 |  | 0 | 0 | 0.0384 | 0.9616 | 0.0523 |  |  |  |  |
|  | | | | | | | | | | | |
| **Histological grade**  **(*n = 90*)**  Grade III  (*n= 31*) | 0 | 500 | 20 | 0 | 1.0000 | 0 | 0 | 0.00129 | 0.000172 | 0.001905 | 0.000375 |
|  | 500 | 1000 | 6 | 0 | 0.3548 | 0.6452 | 0.0859 | 0.000387 | 0.000142 | 0.0015 | 0.000568 |
|  | 1000 | 1500 | 2 | 2 | 0.1613 | 0.8387 | 0.8387 | 0.000161 | 0.000104 | 0.001333 | 0.000889 |
|  | 1500 | 2000 | 0 | 0 | 0.0806 | 0.9194 | 0.9194 |  |  | 0 |  |
|  | 2000 | 2500 | 1 | 0 | 0.0806 | 0.9194 | 0.9194 | 0.000161 | 0.000104 | 0.004 | 0 |
|  | 2500 |  | 0 | 0 | 0 | 1.0000 | 1.0000 |  |  |  |  |
|  | | | | | | | | | | | |
| **Ulceration – Absent**  (*n = 67*) | 0 | 500 | 22 | 7 | 1.0000 | 0 | 0 | 0.000693 | 0.000119 | 0.000838 | 0.000175 |
|  | 500 | 1000 | 10 | 5 | 0.6535 | 0.3465 | 0.0597 | 0.000368 | 0.000104 | 0.000104 | 0.000205 |
|  | 1000 | 1500 | 8 | 10 | 0.4694 | 0.5306 | 0.0654 | 0.000417 | 0.000124 | 0.001143 | 0.000387 |
|  | 1500 | 2000 | 3 | 1 | 0.2608 | 0.7392 | 0.0659 | 0.000348 | 0.000145 | 0.002 | 0.001 |
|  | 2000 | 2500 | 1 | 0 | 0.9131 | 0.9131 | 0.0620 | 0.000174 | 0.000124 | 0.004 | 0 |
|  | 2500 |  | 0 | 0 | 0 | 1.0000 | 0 |  |  |  |  |
|  | | | | | | | | | | | |
| **Ulceration – Present**  (*n = 28*) | 0 | 500 | 17 | 0 | 1.0000 | 0 | 0 | 0.00121 | 0.000185 | 0.001744 | 0.000381 |
|  | 500 | 1000 | 5 | 1 | 0.3929 | 0.6071 | 0.0923 | 0.000374 | 0.000150 | 0.00125 | 0.000531 |
|  | 1000 | 1500 | 4 | 0 | 0.2058 | 0.7942 | 0.0775 | 0.000329 | 0.000144 | 0.002667 | 0.000994 |
|  | 1500 | 2000 | 1 | 0 | 0.0412 | 0.9588 | 0.0399 | 0.000082 | 0.000080 | 0.004 | 0 |
|  | 2000 |  | 0 | 0 | 0 | 1.0000 | 0 |  |  |  |  |
| **Disease-free interval** (*n*=95) | | | | | | | | | | | |
| **Age ≤9.0 years**  (*n = 30)* | 0 | 500 | 12 | 3 | 1.0000 | 0 | 0 | 0.000539 | 0.000133 | 0.000623 | 0.000178 |
|  | 500 | 1000 | 6 | 5 | 0.7303 | 0.2697 | 0.0665 | 0.000308 | 0.000115 | 0.000471 | 0.000191 |
|  | 1000 | 1500 | 9 | 7 | 0.5766 | 0.4234 | 0.0766 | 0.000629 | 0.000164 | 0.0015 | 0.000464 |
|  | 1500 | 2000 | 1 | 2 | 0.2621 | 0.7379 | 0.0788 | 0.000175 | 0.000152 | 0.0008 | 0.000784 |
|  | 2000 | 2500 | 1 | 0 | 0.1747 | 0.8253 | 0.0886 | 0.000349 | 0.000177 | 0.004 | 0 |
|  | 2500 |  | 0 | 0 | 0 | 1.0000 | 0 |  |  |  |  |
|  | | | | | | | | | | | |
| **Age ˃9.0 years**  (*n = 65*) | 0 | 500 | 30 | 4 | 1.0000 | 0 | 0 | 0.000128 | 0.000140 | 0.001875 | 0.000302 |
|  | 500 | 1000 | 6 | 0 | 0.3617 | 0.6383 | 0.0701 | 0.000289 | 0.000107 | 0.001 | 0.000395 |
|  | 1000 | 1500 | 5 | 3 | 0.2170 | 0.7830 | 0.0621 | 0.000289 | 0.000112 | 0.002 | 0.000775 |
|  | 1500 | 2000 | 0 | 0 | 0.0723 | 0.9277 | 0.0427 | 0 |  | 0 |  |
|  | 2000 | 2500 | 1 | 0 | 0.0723 | 0.9277 | 0.0427 | 0.000145 | 0.000085 | 0.004 | 0 |
|  | 2500 |  | 0 | 0 | 0 | 0 | 0 |  |  |  |  |
|  | | | | | | | | | | | |
| **Pseudocyesis –Absent**  (*n = 88*) | 0 | 500 | 41 | 6 | 1.0000 | 0 | 0 | 0.000965 | 0.000108 | 0.001271 | 0.000188 |
|  | 500 | 1000 | 11 | 5 | 0.5176 | 0.4824 | 0.0542 | 0.000296 | 0.000081 | 0.000667 | 0.000198 |
|  | 1000 | 1500 | 14 | 9 | 0.3697 | 0.6303 | 0.0540 | 0.000505 | 0.000106 | 0.002074 | 0.000474 |
|  | 1500 | 2000 | 0 | 1 | 0.1172 | 0.8828 | 0.0417 | 0 |  | 0 |  |
|  | 2000 | 2500 | 1 | 0 | 0.1172 | 0.8828 | 0.0417 | 0.000234 | 0.000083 | 0.004 | 0 |
|  | 2500 |  | 0 | 0 | 0 | 1.0000 | 0 |  |  |  |  |
|  | | | | | | | | | | | |
| **Pseudocyesis –Present**  (*n = 7*) | 0 | 500 | 1 | 1 | 1.0000 | 0 | 0 | 0.000308 | 0.000283 | 0.000333 | 0.000332 |
|  | 500 | 1000 | 1 | 0 | 0.8462 | 0.1538 | 0.1415 | 0.000338 | 0.000308 | 0.000444 | 0.000442 |
|  | 1000 | 1500 | 0 | 1 | 0.6769 | 0.3231 | 0.1890 | 0 |  | 0 |  |
|  | 1500 | 2000 | 1 | 1 | 0.6769 | 0.3231 | 0.1890 | 0.000542 | 0.000446 | 0.001 | 0.000968 |
|  | 2000 | 2500 | 1 | 0 | 0.4062 | 0.5938 | 0.2384 | 0.000812 | 0.000477 | 0.004 | 0 |
|  | 2500 |  | 0 | 0 | 0 | 1.0000 | 0 |  |  |  |  |
|  | | | | | | | | | | | |
| **Clinical Stage** (TNM)  Initial (I–III)  (*n= 74*) | 0 | 500 | 29 | 7 | 1.0000 | 0 | 0 | 0.000823 | 0.000117 | 0.001036 | 0.000186 |
|  | 500 | 1000 | 7 | 4 | 0.5887 | 0.4113 | 0.0586 | 0.000229 | 0.000081 | 0.000431 | 0.000162 |
|  | 1000 | 1500 | 13 | 9 | 0.4742 | 0.5258 | 0.0611 | 0.000548 | 0.000121 | 0.001625 | 0.000412 |
|  | 1500 | 2000 | 1 | 2 | 0.2002 | 0.7998 | 0.0557 | 0.000100 | 0.000091 | 0.000571 | 0.000566 |
|  | 2000 | 2500 | 2 | 0 | 0.1502 | 0.8498 | 0.0602 | 0.000300 | 0.000120 | 0.004 | 0 |
|  | 2500 |  | 0 | 0 | 0 | 1.0000 | 0 |  |  |  |  |
|  | | | | | | | | | | | |
| **Clinical Stage** (TNM)  Advanced (IV–V)  (*n=21* ) | 0 | 500 | 13 | 0 | 1.0000 | 0 | 0 | 0.00124 | 0.000212 | 0.001793 | 0.000445 |
|  | 500 | 1000 | 5 | 1 | 0.3810 | 0.6190 | 0.1060 | 0.000508 | 0.000193 | 0.002 | 0.000775 |
|  | 1000 | 1500 | 1 | 1 | 0.1270 | 0.8730 | 0.0745 | 0.000169 | 0.000139 | 0.002 | 0.001732 |
|  | 1500 |  | 0 | 0 | 0.0423 | 0.9577 | 0.0548 |  |  |  |  |
|  | | | | | | | | | | | |
| **Histological grade**  **(*n = 90*)**  Grade I  (*n= 32*) | 0 | 500 | 6 | 6 | 1.0000 | 0 | 0 | 0.000414 | 0.000150 | 0.000462 | 0.000187 |
|  | 500 | 1000 | 4 | 3 | 0.7931 | 0.2069 | 0.0752 | 0.000343 | 0.000155 | 0.000485 | 0.000241 |
|  | 1000 | 1500 | 6 | 6 | 0.6216 | 0.3784 | 0.0961 | 0.000746 | 0.000224 | 0.001714 | 0.000632 |
|  | 1500 | 2000 | 1 | 0 | 0.2486 | 0.7514 | 0.1037 | 0.000497 | 0.000207 | 0.004 | 0 |
|  | 2000 |  | 0 | 0 | 0 | 1.0000 | 0 |  |  |  |  |
|  | | | | | | | | | | | |
| **Histological grade**  **(*n = 90*)**  Grade II  (*n= 27*) | 0 | 500 | 11 | 1 | 1.0000 | 0 | 0 | 0.000830 | 0.000191 | 0.001048 | 0.000305 |
|  | 500 | 1000 | 2 | 2 | 0.5849 | 0.4151 | 0.0957 | 0.000167 | 0.000113 | 0.000308 | 0.000217 |
|  | 1000 | 1500 | 6 | 2 | 0.5013 | 0.4987 | 0.0986 | 0.000602 | 0.000195 | 0.001714 | 0.000632 |
|  | 1500 | 2000 | 0 | 2 | 0.2005 | 0.7995 | 0.0871 | 0 |  | 0 |  |
|  | 2000 | 2500 | 1 | 0 | 0.2005 | 0.7995 | 0.0871 | 0.000401 | 0.000174 | 0.004 | 0 |
|  | 2500 |  | 0 | 0 | 0 | 1.0000 | 0 |  |  |  |  |
|  | | | | | | | | | | | |
| **Histological grade**  **(*n = 90*)**  Grade III  (*n= 31*) | 0 | 500 | 22 | 0 | 1.0000 | 0 | 0 | 0.00142 | 0.000163 | 0.0022 | 0.000392 |
|  | 500 | 1000 | 4 | 0 | 0.2903 | 0.7097 | 0.0815 | 0.000258 | 0.000120 | 0.001143 | 0.000548 |
|  | 1000 | 1500 | 2 | 2 | 0.1613 | 0.8387 | 0.0661 | 0.000161 | 0.000104 | 0.001333 | 0.000889 |
|  | 1500 | 2000 | 0 | 0 | 0.0806 | 0.9194 | 0.0521 |  |  | 0 |  |
|  | 2000 | 2500 | 1 | 0 | 0.0806 | 0.9194 | 0.0521 | 0.000161 | 0.000104 | 0.004 | 0 |
|  | 2500 |  | 0 | 0 | 0 | 1.0000 | 0 |  |  |  |  |
|  | | | | | | | | | | | |
| **Ulceration – Absent**  (*n = 67*) | 0 | 500 | 23 | 7 | 1.0000 | 0 | 0 | 0.000724 | 0.000121 | 0.000885 | 0.00018 |
|  | 500 | 1000 | 8 | 4 | 0.6378 | 0.3622 | 0.0603 | 0.000292 | 0.000095 | 0.000516 | 0.000181 |
|  | 1000 | 1500 | 10 | 10 | 0.4920 | 0.5080 | 0.0649 | 0.000492 | 0.000128 | 0.001333 | 0.000398 |
|  | 1500 | 2000 | 1 | 2 | 0.2460 | 0.7540 | 0.0639 | 0.000123 | 0.000111 | 0.000571 | 0.000566 |
|  | 2000 | 2500 | 2 | 0 | 0.1845 | 0.8155 | 0.0716 | 0.000369 | 0.000143 | 0.004 | 0 |
|  | 2500 |  | 0 | 0 | 0 | 1.0000 | 0 |  |  |  |  |
|  | | | | | | | | | | | |
| **Ulceration – Present**  (*n = 28*) | 0 | 500 | 19 | 0 | 1.0000 | 0 | 0 | 0.00136 | 0.000177 | 0.002054 | 0.000404 |
|  | 500 | 1000 | 4 | 1 | 0.3214 | 0.6786 | 0.0883 | 0.000303 | 0.000138 | 0.001231 | 0.000586 |
|  | 1000 | 1500 | 4 | 0 | 0.1702 | 0.8298 | 0.0722 | 0.000340 | 0.000144 | 0.004 | 0 |
|  | 1500 |  | 0 | 0 | 0 | 1.0000 | 0 |  |  |  |  |
