# Supplementary figures and images for: Prognostic Factors for Cancer-Specific Survival and Disease-Free Interval of Dogs with Mammary Carcinomas
Source: Vet Med Int. 2023 Aug 4;2023:6890707. doi: 10.1155/2023/6890707 (PMC10421712; doi:10.1155/2023/6890707)

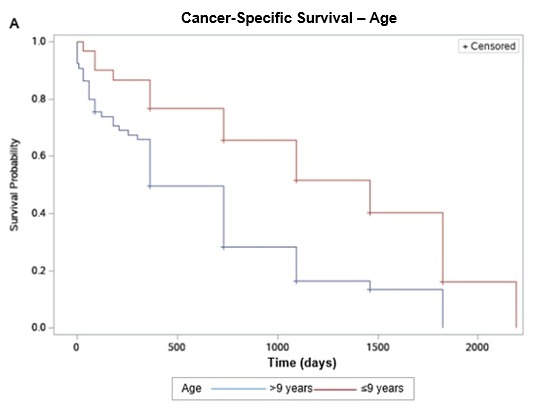

Supplement: Supplementary Materials — Supplementary Figure 1: cancer-specific survival and disease-free interval Kaplan–Meier curve of female dogs with malignant mammary neoplasms. Malignant mammary neoplasms are classified according to (A) CSS age: ≤9 years (median: 1.460 days) and >9 years (median: 365 days); (B) DFI age: ≤9 years (median: 1.460 days) and >9 years (median: 365 days); (C) DFI pseudocyesis: present (median: 1.825 days) and absent (median: 730 days); (D) CSS disease history: negative (median: 730 days) and positive (median: 547 days); (E) CSS clinical staging: initial (median: 730 days) and advanced (365 days); (F) DFI clinical staging: initial (median: 730 days) and advanced (365 days); (G) CSS histological grade: I (median: 1.095 days), II (1.095 days), and III (365 days), respectively; (H) DFI histological grade: I (median: 1.095 days), II (1.095 days), and III (180 days), respectively; (I) CSS ulceration: present (median: 365 days) and absent (median: 730 days); (J) DFI ulceration: present (median: 365 days) and absent (median: 730 days). Supplementary Figure 2: overall survival Kaplan–Meier curve of female dogs with malignant mammary neoplasms. Malignant mammary neoplasms are classified according to (A) age: ≤9 years (median: 1.095 days) and >9 years (median: 365 days); (B) pseudocyesis: present (median: 1.095 days) and absent (median: 730 days); (C) clinical staging: initial (median: 730 days) and advanced (365 days); (D) histological grade: I (median: 1.095 days), II (730 days), and III (365 days), respectively; (E) ulceration: present (median: 730 days) and absent (median: 730 days). Supplementary Table 1: histopathological classification and frequency in % of 385 cases of mammary tumors diagnosed in female dogs treated at the UFV Veterinary Hospital, which were classified into non-neoplastic lesions (n = 27/7.01%), benign neoplasms (n = 16/4.16%), and malignant neoplasms (n = 342/88.83%). Supplementary Table 2: estimates of life survival and risk functions in 95 cases with avail [file 6890707.f1.zip › 6890707.f1/Supplementary -Figure 1A.jpeg]

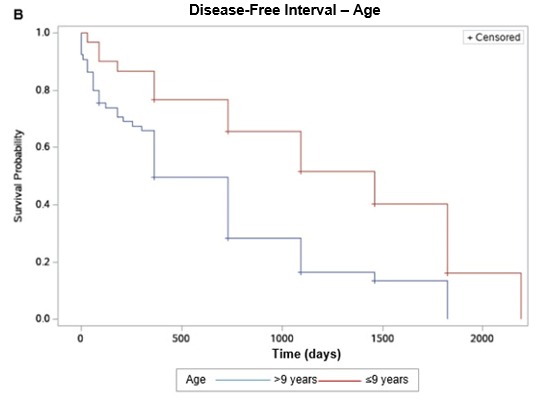

Supplement: Supplementary Materials — Supplementary Figure 1: cancer-specific survival and disease-free interval Kaplan–Meier curve of female dogs with malignant mammary neoplasms. Malignant mammary neoplasms are classified according to (A) CSS age: ≤9 years (median: 1.460 days) and >9 years (median: 365 days); (B) DFI age: ≤9 years (median: 1.460 days) and >9 years (median: 365 days); (C) DFI pseudocyesis: present (median: 1.825 days) and absent (median: 730 days); (D) CSS disease history: negative (median: 730 days) and positive (median: 547 days); (E) CSS clinical staging: initial (median: 730 days) and advanced (365 days); (F) DFI clinical staging: initial (median: 730 days) and advanced (365 days); (G) CSS histological grade: I (median: 1.095 days), II (1.095 days), and III (365 days), respectively; (H) DFI histological grade: I (median: 1.095 days), II (1.095 days), and III (180 days), respectively; (I) CSS ulceration: present (median: 365 days) and absent (median: 730 days); (J) DFI ulceration: present (median: 365 days) and absent (median: 730 days). Supplementary Figure 2: overall survival Kaplan–Meier curve of female dogs with malignant mammary neoplasms. Malignant mammary neoplasms are classified according to (A) age: ≤9 years (median: 1.095 days) and >9 years (median: 365 days); (B) pseudocyesis: present (median: 1.095 days) and absent (median: 730 days); (C) clinical staging: initial (median: 730 days) and advanced (365 days); (D) histological grade: I (median: 1.095 days), II (730 days), and III (365 days), respectively; (E) ulceration: present (median: 730 days) and absent (median: 730 days). Supplementary Table 1: histopathological classification and frequency in % of 385 cases of mammary tumors diagnosed in female dogs treated at the UFV Veterinary Hospital, which were classified into non-neoplastic lesions (n = 27/7.01%), benign neoplasms (n = 16/4.16%), and malignant neoplasms (n = 342/88.83%). Supplementary Table 2: estimates of life survival and risk functions in 95 cases with avail [file 6890707.f1.zip › 6890707.f1/Supplementary -Figure 1B.jpeg]

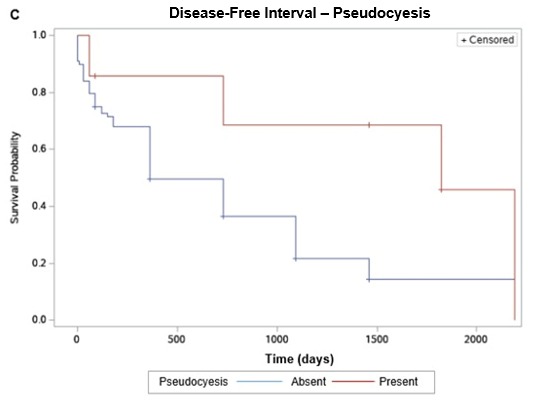

Supplement: Supplementary Materials — Supplementary Figure 1: cancer-specific survival and disease-free interval Kaplan–Meier curve of female dogs with malignant mammary neoplasms. Malignant mammary neoplasms are classified according to (A) CSS age: ≤9 years (median: 1.460 days) and >9 years (median: 365 days); (B) DFI age: ≤9 years (median: 1.460 days) and >9 years (median: 365 days); (C) DFI pseudocyesis: present (median: 1.825 days) and absent (median: 730 days); (D) CSS disease history: negative (median: 730 days) and positive (median: 547 days); (E) CSS clinical staging: initial (median: 730 days) and advanced (365 days); (F) DFI clinical staging: initial (median: 730 days) and advanced (365 days); (G) CSS histological grade: I (median: 1.095 days), II (1.095 days), and III (365 days), respectively; (H) DFI histological grade: I (median: 1.095 days), II (1.095 days), and III (180 days), respectively; (I) CSS ulceration: present (median: 365 days) and absent (median: 730 days); (J) DFI ulceration: present (median: 365 days) and absent (median: 730 days). Supplementary Figure 2: overall survival Kaplan–Meier curve of female dogs with malignant mammary neoplasms. Malignant mammary neoplasms are classified according to (A) age: ≤9 years (median: 1.095 days) and >9 years (median: 365 days); (B) pseudocyesis: present (median: 1.095 days) and absent (median: 730 days); (C) clinical staging: initial (median: 730 days) and advanced (365 days); (D) histological grade: I (median: 1.095 days), II (730 days), and III (365 days), respectively; (E) ulceration: present (median: 730 days) and absent (median: 730 days). Supplementary Table 1: histopathological classification and frequency in % of 385 cases of mammary tumors diagnosed in female dogs treated at the UFV Veterinary Hospital, which were classified into non-neoplastic lesions (n = 27/7.01%), benign neoplasms (n = 16/4.16%), and malignant neoplasms (n = 342/88.83%). Supplementary Table 2: estimates of life survival and risk functions in 95 cases with avail [file 6890707.f1.zip › 6890707.f1/Supplementary -Figure 1C.jpeg]

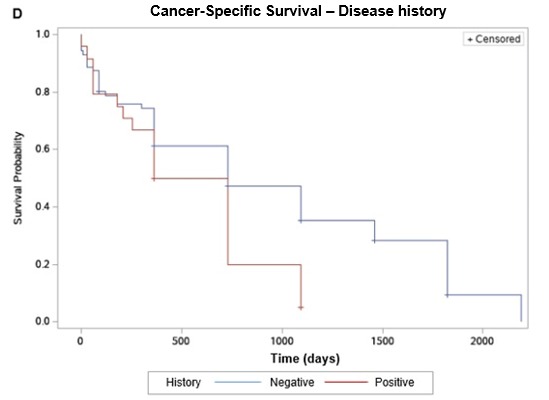

Supplement: Supplementary Materials — Supplementary Figure 1: cancer-specific survival and disease-free interval Kaplan–Meier curve of female dogs with malignant mammary neoplasms. Malignant mammary neoplasms are classified according to (A) CSS age: ≤9 years (median: 1.460 days) and >9 years (median: 365 days); (B) DFI age: ≤9 years (median: 1.460 days) and >9 years (median: 365 days); (C) DFI pseudocyesis: present (median: 1.825 days) and absent (median: 730 days); (D) CSS disease history: negative (median: 730 days) and positive (median: 547 days); (E) CSS clinical staging: initial (median: 730 days) and advanced (365 days); (F) DFI clinical staging: initial (median: 730 days) and advanced (365 days); (G) CSS histological grade: I (median: 1.095 days), II (1.095 days), and III (365 days), respectively; (H) DFI histological grade: I (median: 1.095 days), II (1.095 days), and III (180 days), respectively; (I) CSS ulceration: present (median: 365 days) and absent (median: 730 days); (J) DFI ulceration: present (median: 365 days) and absent (median: 730 days). Supplementary Figure 2: overall survival Kaplan–Meier curve of female dogs with malignant mammary neoplasms. Malignant mammary neoplasms are classified according to (A) age: ≤9 years (median: 1.095 days) and >9 years (median: 365 days); (B) pseudocyesis: present (median: 1.095 days) and absent (median: 730 days); (C) clinical staging: initial (median: 730 days) and advanced (365 days); (D) histological grade: I (median: 1.095 days), II (730 days), and III (365 days), respectively; (E) ulceration: present (median: 730 days) and absent (median: 730 days). Supplementary Table 1: histopathological classification and frequency in % of 385 cases of mammary tumors diagnosed in female dogs treated at the UFV Veterinary Hospital, which were classified into non-neoplastic lesions (n = 27/7.01%), benign neoplasms (n = 16/4.16%), and malignant neoplasms (n = 342/88.83%). Supplementary Table 2: estimates of life survival and risk functions in 95 cases with avail [file 6890707.f1.zip › 6890707.f1/Supplementary -Figure 1D.jpeg]

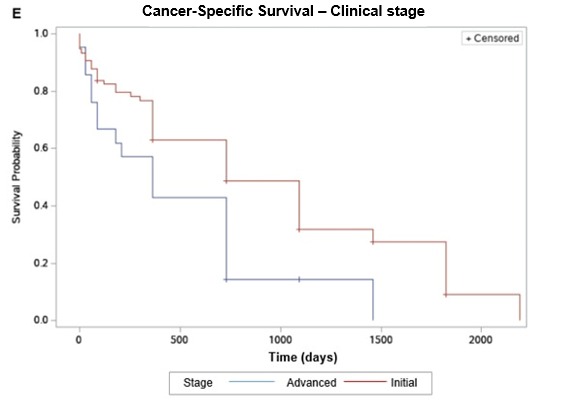

Supplement: Supplementary Materials — Supplementary Figure 1: cancer-specific survival and disease-free interval Kaplan–Meier curve of female dogs with malignant mammary neoplasms. Malignant mammary neoplasms are classified according to (A) CSS age: ≤9 years (median: 1.460 days) and >9 years (median: 365 days); (B) DFI age: ≤9 years (median: 1.460 days) and >9 years (median: 365 days); (C) DFI pseudocyesis: present (median: 1.825 days) and absent (median: 730 days); (D) CSS disease history: negative (median: 730 days) and positive (median: 547 days); (E) CSS clinical staging: initial (median: 730 days) and advanced (365 days); (F) DFI clinical staging: initial (median: 730 days) and advanced (365 days); (G) CSS histological grade: I (median: 1.095 days), II (1.095 days), and III (365 days), respectively; (H) DFI histological grade: I (median: 1.095 days), II (1.095 days), and III (180 days), respectively; (I) CSS ulceration: present (median: 365 days) and absent (median: 730 days); (J) DFI ulceration: present (median: 365 days) and absent (median: 730 days). Supplementary Figure 2: overall survival Kaplan–Meier curve of female dogs with malignant mammary neoplasms. Malignant mammary neoplasms are classified according to (A) age: ≤9 years (median: 1.095 days) and >9 years (median: 365 days); (B) pseudocyesis: present (median: 1.095 days) and absent (median: 730 days); (C) clinical staging: initial (median: 730 days) and advanced (365 days); (D) histological grade: I (median: 1.095 days), II (730 days), and III (365 days), respectively; (E) ulceration: present (median: 730 days) and absent (median: 730 days). Supplementary Table 1: histopathological classification and frequency in % of 385 cases of mammary tumors diagnosed in female dogs treated at the UFV Veterinary Hospital, which were classified into non-neoplastic lesions (n = 27/7.01%), benign neoplasms (n = 16/4.16%), and malignant neoplasms (n = 342/88.83%). Supplementary Table 2: estimates of life survival and risk functions in 95 cases with avail [file 6890707.f1.zip › 6890707.f1/Supplementary -Figure 1E.jpeg]

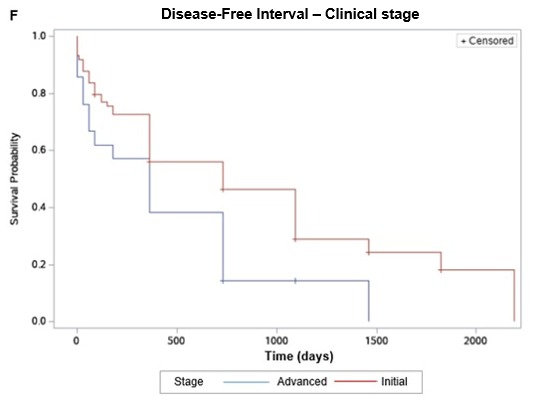

Supplement: Supplementary Materials — Supplementary Figure 1: cancer-specific survival and disease-free interval Kaplan–Meier curve of female dogs with malignant mammary neoplasms. Malignant mammary neoplasms are classified according to (A) CSS age: ≤9 years (median: 1.460 days) and >9 years (median: 365 days); (B) DFI age: ≤9 years (median: 1.460 days) and >9 years (median: 365 days); (C) DFI pseudocyesis: present (median: 1.825 days) and absent (median: 730 days); (D) CSS disease history: negative (median: 730 days) and positive (median: 547 days); (E) CSS clinical staging: initial (median: 730 days) and advanced (365 days); (F) DFI clinical staging: initial (median: 730 days) and advanced (365 days); (G) CSS histological grade: I (median: 1.095 days), II (1.095 days), and III (365 days), respectively; (H) DFI histological grade: I (median: 1.095 days), II (1.095 days), and III (180 days), respectively; (I) CSS ulceration: present (median: 365 days) and absent (median: 730 days); (J) DFI ulceration: present (median: 365 days) and absent (median: 730 days). Supplementary Figure 2: overall survival Kaplan–Meier curve of female dogs with malignant mammary neoplasms. Malignant mammary neoplasms are classified according to (A) age: ≤9 years (median: 1.095 days) and >9 years (median: 365 days); (B) pseudocyesis: present (median: 1.095 days) and absent (median: 730 days); (C) clinical staging: initial (median: 730 days) and advanced (365 days); (D) histological grade: I (median: 1.095 days), II (730 days), and III (365 days), respectively; (E) ulceration: present (median: 730 days) and absent (median: 730 days). Supplementary Table 1: histopathological classification and frequency in % of 385 cases of mammary tumors diagnosed in female dogs treated at the UFV Veterinary Hospital, which were classified into non-neoplastic lesions (n = 27/7.01%), benign neoplasms (n = 16/4.16%), and malignant neoplasms (n = 342/88.83%). Supplementary Table 2: estimates of life survival and risk functions in 95 cases with avail [file 6890707.f1.zip › 6890707.f1/Supplementary -Figure 1F.jpeg]

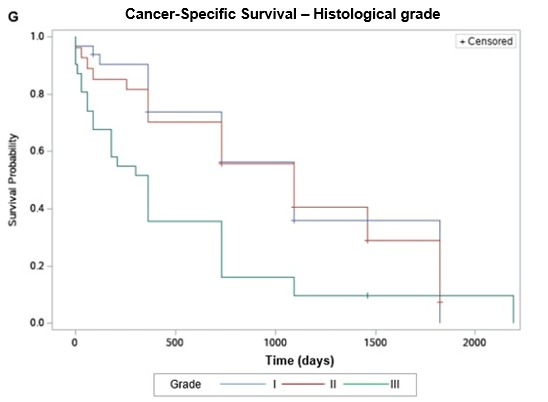

Supplement: Supplementary Materials — Supplementary Figure 1: cancer-specific survival and disease-free interval Kaplan–Meier curve of female dogs with malignant mammary neoplasms. Malignant mammary neoplasms are classified according to (A) CSS age: ≤9 years (median: 1.460 days) and >9 years (median: 365 days); (B) DFI age: ≤9 years (median: 1.460 days) and >9 years (median: 365 days); (C) DFI pseudocyesis: present (median: 1.825 days) and absent (median: 730 days); (D) CSS disease history: negative (median: 730 days) and positive (median: 547 days); (E) CSS clinical staging: initial (median: 730 days) and advanced (365 days); (F) DFI clinical staging: initial (median: 730 days) and advanced (365 days); (G) CSS histological grade: I (median: 1.095 days), II (1.095 days), and III (365 days), respectively; (H) DFI histological grade: I (median: 1.095 days), II (1.095 days), and III (180 days), respectively; (I) CSS ulceration: present (median: 365 days) and absent (median: 730 days); (J) DFI ulceration: present (median: 365 days) and absent (median: 730 days). Supplementary Figure 2: overall survival Kaplan–Meier curve of female dogs with malignant mammary neoplasms. Malignant mammary neoplasms are classified according to (A) age: ≤9 years (median: 1.095 days) and >9 years (median: 365 days); (B) pseudocyesis: present (median: 1.095 days) and absent (median: 730 days); (C) clinical staging: initial (median: 730 days) and advanced (365 days); (D) histological grade: I (median: 1.095 days), II (730 days), and III (365 days), respectively; (E) ulceration: present (median: 730 days) and absent (median: 730 days). Supplementary Table 1: histopathological classification and frequency in % of 385 cases of mammary tumors diagnosed in female dogs treated at the UFV Veterinary Hospital, which were classified into non-neoplastic lesions (n = 27/7.01%), benign neoplasms (n = 16/4.16%), and malignant neoplasms (n = 342/88.83%). Supplementary Table 2: estimates of life survival and risk functions in 95 cases with avail [file 6890707.f1.zip › 6890707.f1/Supplementary -Figure 1G.jpeg]

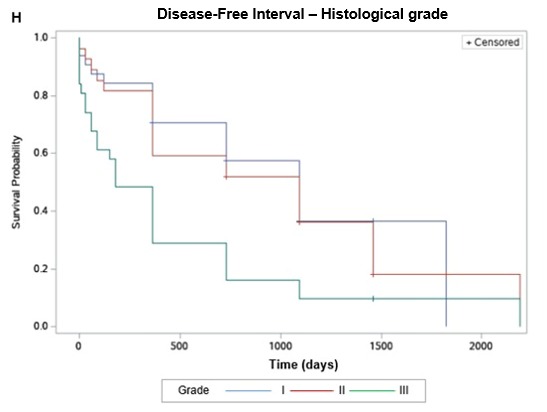

Supplement: Supplementary Materials — Supplementary Figure 1: cancer-specific survival and disease-free interval Kaplan–Meier curve of female dogs with malignant mammary neoplasms. Malignant mammary neoplasms are classified according to (A) CSS age: ≤9 years (median: 1.460 days) and >9 years (median: 365 days); (B) DFI age: ≤9 years (median: 1.460 days) and >9 years (median: 365 days); (C) DFI pseudocyesis: present (median: 1.825 days) and absent (median: 730 days); (D) CSS disease history: negative (median: 730 days) and positive (median: 547 days); (E) CSS clinical staging: initial (median: 730 days) and advanced (365 days); (F) DFI clinical staging: initial (median: 730 days) and advanced (365 days); (G) CSS histological grade: I (median: 1.095 days), II (1.095 days), and III (365 days), respectively; (H) DFI histological grade: I (median: 1.095 days), II (1.095 days), and III (180 days), respectively; (I) CSS ulceration: present (median: 365 days) and absent (median: 730 days); (J) DFI ulceration: present (median: 365 days) and absent (median: 730 days). Supplementary Figure 2: overall survival Kaplan–Meier curve of female dogs with malignant mammary neoplasms. Malignant mammary neoplasms are classified according to (A) age: ≤9 years (median: 1.095 days) and >9 years (median: 365 days); (B) pseudocyesis: present (median: 1.095 days) and absent (median: 730 days); (C) clinical staging: initial (median: 730 days) and advanced (365 days); (D) histological grade: I (median: 1.095 days), II (730 days), and III (365 days), respectively; (E) ulceration: present (median: 730 days) and absent (median: 730 days). Supplementary Table 1: histopathological classification and frequency in % of 385 cases of mammary tumors diagnosed in female dogs treated at the UFV Veterinary Hospital, which were classified into non-neoplastic lesions (n = 27/7.01%), benign neoplasms (n = 16/4.16%), and malignant neoplasms (n = 342/88.83%). Supplementary Table 2: estimates of life survival and risk functions in 95 cases with avail [file 6890707.f1.zip › 6890707.f1/Supplementary -Figure 1H.jpeg]

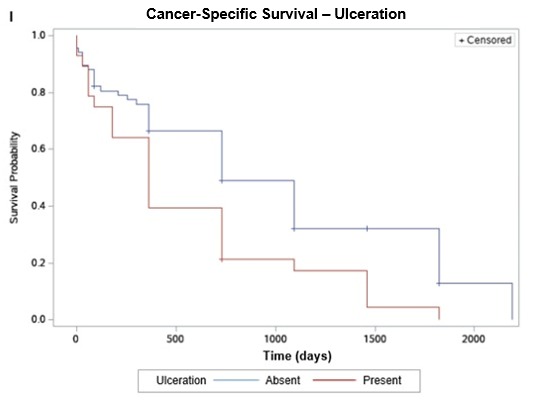

Supplement: Supplementary Materials — Supplementary Figure 1: cancer-specific survival and disease-free interval Kaplan–Meier curve of female dogs with malignant mammary neoplasms. Malignant mammary neoplasms are classified according to (A) CSS age: ≤9 years (median: 1.460 days) and >9 years (median: 365 days); (B) DFI age: ≤9 years (median: 1.460 days) and >9 years (median: 365 days); (C) DFI pseudocyesis: present (median: 1.825 days) and absent (median: 730 days); (D) CSS disease history: negative (median: 730 days) and positive (median: 547 days); (E) CSS clinical staging: initial (median: 730 days) and advanced (365 days); (F) DFI clinical staging: initial (median: 730 days) and advanced (365 days); (G) CSS histological grade: I (median: 1.095 days), II (1.095 days), and III (365 days), respectively; (H) DFI histological grade: I (median: 1.095 days), II (1.095 days), and III (180 days), respectively; (I) CSS ulceration: present (median: 365 days) and absent (median: 730 days); (J) DFI ulceration: present (median: 365 days) and absent (median: 730 days). Supplementary Figure 2: overall survival Kaplan–Meier curve of female dogs with malignant mammary neoplasms. Malignant mammary neoplasms are classified according to (A) age: ≤9 years (median: 1.095 days) and >9 years (median: 365 days); (B) pseudocyesis: present (median: 1.095 days) and absent (median: 730 days); (C) clinical staging: initial (median: 730 days) and advanced (365 days); (D) histological grade: I (median: 1.095 days), II (730 days), and III (365 days), respectively; (E) ulceration: present (median: 730 days) and absent (median: 730 days). Supplementary Table 1: histopathological classification and frequency in % of 385 cases of mammary tumors diagnosed in female dogs treated at the UFV Veterinary Hospital, which were classified into non-neoplastic lesions (n = 27/7.01%), benign neoplasms (n = 16/4.16%), and malignant neoplasms (n = 342/88.83%). Supplementary Table 2: estimates of life survival and risk functions in 95 cases with avail [file 6890707.f1.zip › 6890707.f1/Supplementary -Figure 1I.jpeg]

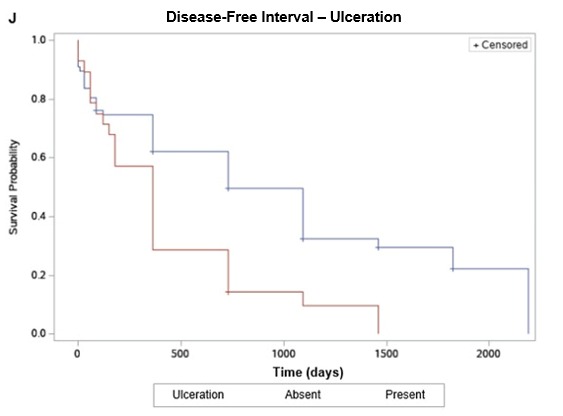

Supplement: Supplementary Materials — Supplementary Figure 1: cancer-specific survival and disease-free interval Kaplan–Meier curve of female dogs with malignant mammary neoplasms. Malignant mammary neoplasms are classified according to (A) CSS age: ≤9 years (median: 1.460 days) and >9 years (median: 365 days); (B) DFI age: ≤9 years (median: 1.460 days) and >9 years (median: 365 days); (C) DFI pseudocyesis: present (median: 1.825 days) and absent (median: 730 days); (D) CSS disease history: negative (median: 730 days) and positive (median: 547 days); (E) CSS clinical staging: initial (median: 730 days) and advanced (365 days); (F) DFI clinical staging: initial (median: 730 days) and advanced (365 days); (G) CSS histological grade: I (median: 1.095 days), II (1.095 days), and III (365 days), respectively; (H) DFI histological grade: I (median: 1.095 days), II (1.095 days), and III (180 days), respectively; (I) CSS ulceration: present (median: 365 days) and absent (median: 730 days); (J) DFI ulceration: present (median: 365 days) and absent (median: 730 days). Supplementary Figure 2: overall survival Kaplan–Meier curve of female dogs with malignant mammary neoplasms. Malignant mammary neoplasms are classified according to (A) age: ≤9 years (median: 1.095 days) and >9 years (median: 365 days); (B) pseudocyesis: present (median: 1.095 days) and absent (median: 730 days); (C) clinical staging: initial (median: 730 days) and advanced (365 days); (D) histological grade: I (median: 1.095 days), II (730 days), and III (365 days), respectively; (E) ulceration: present (median: 730 days) and absent (median: 730 days). Supplementary Table 1: histopathological classification and frequency in % of 385 cases of mammary tumors diagnosed in female dogs treated at the UFV Veterinary Hospital, which were classified into non-neoplastic lesions (n = 27/7.01%), benign neoplasms (n = 16/4.16%), and malignant neoplasms (n = 342/88.83%). Supplementary Table 2: estimates of life survival and risk functions in 95 cases with avail [file 6890707.f1.zip › 6890707.f1/Supplementary -Figure 1J (1).jpeg]

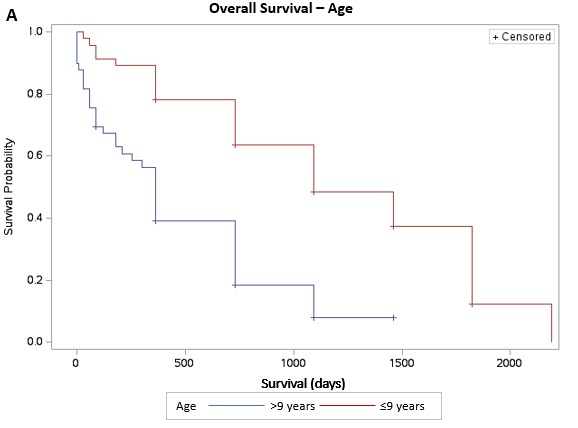

Supplement: Supplementary Materials — Supplementary Figure 1: cancer-specific survival and disease-free interval Kaplan–Meier curve of female dogs with malignant mammary neoplasms. Malignant mammary neoplasms are classified according to (A) CSS age: ≤9 years (median: 1.460 days) and >9 years (median: 365 days); (B) DFI age: ≤9 years (median: 1.460 days) and >9 years (median: 365 days); (C) DFI pseudocyesis: present (median: 1.825 days) and absent (median: 730 days); (D) CSS disease history: negative (median: 730 days) and positive (median: 547 days); (E) CSS clinical staging: initial (median: 730 days) and advanced (365 days); (F) DFI clinical staging: initial (median: 730 days) and advanced (365 days); (G) CSS histological grade: I (median: 1.095 days), II (1.095 days), and III (365 days), respectively; (H) DFI histological grade: I (median: 1.095 days), II (1.095 days), and III (180 days), respectively; (I) CSS ulceration: present (median: 365 days) and absent (median: 730 days); (J) DFI ulceration: present (median: 365 days) and absent (median: 730 days). Supplementary Figure 2: overall survival Kaplan–Meier curve of female dogs with malignant mammary neoplasms. Malignant mammary neoplasms are classified according to (A) age: ≤9 years (median: 1.095 days) and >9 years (median: 365 days); (B) pseudocyesis: present (median: 1.095 days) and absent (median: 730 days); (C) clinical staging: initial (median: 730 days) and advanced (365 days); (D) histological grade: I (median: 1.095 days), II (730 days), and III (365 days), respectively; (E) ulceration: present (median: 730 days) and absent (median: 730 days). Supplementary Table 1: histopathological classification and frequency in % of 385 cases of mammary tumors diagnosed in female dogs treated at the UFV Veterinary Hospital, which were classified into non-neoplastic lesions (n = 27/7.01%), benign neoplasms (n = 16/4.16%), and malignant neoplasms (n = 342/88.83%). Supplementary Table 2: estimates of life survival and risk functions in 95 cases with avail [file 6890707.f1.zip › 6890707.f1/Supplementary -Figure 2A.jpeg]

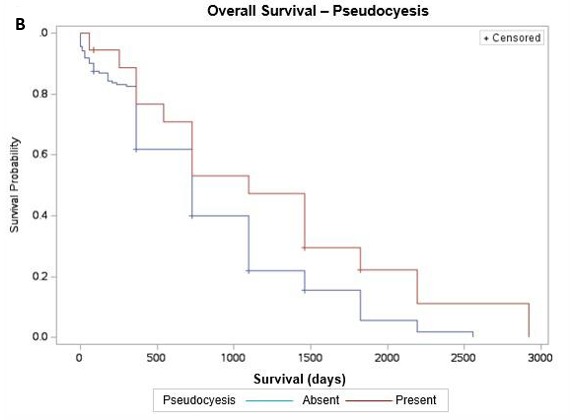

Supplement: Supplementary Materials — Supplementary Figure 1: cancer-specific survival and disease-free interval Kaplan–Meier curve of female dogs with malignant mammary neoplasms. Malignant mammary neoplasms are classified according to (A) CSS age: ≤9 years (median: 1.460 days) and >9 years (median: 365 days); (B) DFI age: ≤9 years (median: 1.460 days) and >9 years (median: 365 days); (C) DFI pseudocyesis: present (median: 1.825 days) and absent (median: 730 days); (D) CSS disease history: negative (median: 730 days) and positive (median: 547 days); (E) CSS clinical staging: initial (median: 730 days) and advanced (365 days); (F) DFI clinical staging: initial (median: 730 days) and advanced (365 days); (G) CSS histological grade: I (median: 1.095 days), II (1.095 days), and III (365 days), respectively; (H) DFI histological grade: I (median: 1.095 days), II (1.095 days), and III (180 days), respectively; (I) CSS ulceration: present (median: 365 days) and absent (median: 730 days); (J) DFI ulceration: present (median: 365 days) and absent (median: 730 days). Supplementary Figure 2: overall survival Kaplan–Meier curve of female dogs with malignant mammary neoplasms. Malignant mammary neoplasms are classified according to (A) age: ≤9 years (median: 1.095 days) and >9 years (median: 365 days); (B) pseudocyesis: present (median: 1.095 days) and absent (median: 730 days); (C) clinical staging: initial (median: 730 days) and advanced (365 days); (D) histological grade: I (median: 1.095 days), II (730 days), and III (365 days), respectively; (E) ulceration: present (median: 730 days) and absent (median: 730 days). Supplementary Table 1: histopathological classification and frequency in % of 385 cases of mammary tumors diagnosed in female dogs treated at the UFV Veterinary Hospital, which were classified into non-neoplastic lesions (n = 27/7.01%), benign neoplasms (n = 16/4.16%), and malignant neoplasms (n = 342/88.83%). Supplementary Table 2: estimates of life survival and risk functions in 95 cases with avail [file 6890707.f1.zip › 6890707.f1/Supplementary -Figure 2B.jpeg]

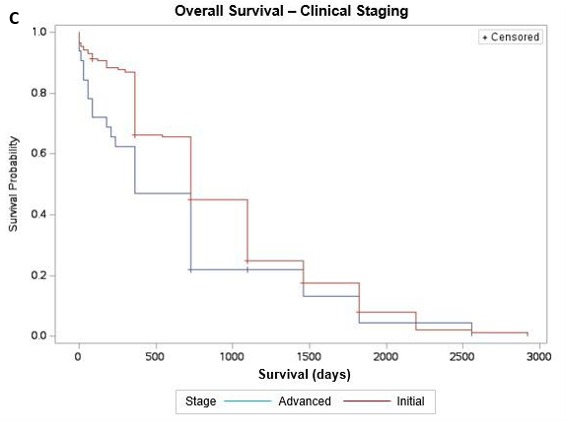

Supplement: Supplementary Materials — Supplementary Figure 1: cancer-specific survival and disease-free interval Kaplan–Meier curve of female dogs with malignant mammary neoplasms. Malignant mammary neoplasms are classified according to (A) CSS age: ≤9 years (median: 1.460 days) and >9 years (median: 365 days); (B) DFI age: ≤9 years (median: 1.460 days) and >9 years (median: 365 days); (C) DFI pseudocyesis: present (median: 1.825 days) and absent (median: 730 days); (D) CSS disease history: negative (median: 730 days) and positive (median: 547 days); (E) CSS clinical staging: initial (median: 730 days) and advanced (365 days); (F) DFI clinical staging: initial (median: 730 days) and advanced (365 days); (G) CSS histological grade: I (median: 1.095 days), II (1.095 days), and III (365 days), respectively; (H) DFI histological grade: I (median: 1.095 days), II (1.095 days), and III (180 days), respectively; (I) CSS ulceration: present (median: 365 days) and absent (median: 730 days); (J) DFI ulceration: present (median: 365 days) and absent (median: 730 days). Supplementary Figure 2: overall survival Kaplan–Meier curve of female dogs with malignant mammary neoplasms. Malignant mammary neoplasms are classified according to (A) age: ≤9 years (median: 1.095 days) and >9 years (median: 365 days); (B) pseudocyesis: present (median: 1.095 days) and absent (median: 730 days); (C) clinical staging: initial (median: 730 days) and advanced (365 days); (D) histological grade: I (median: 1.095 days), II (730 days), and III (365 days), respectively; (E) ulceration: present (median: 730 days) and absent (median: 730 days). Supplementary Table 1: histopathological classification and frequency in % of 385 cases of mammary tumors diagnosed in female dogs treated at the UFV Veterinary Hospital, which were classified into non-neoplastic lesions (n = 27/7.01%), benign neoplasms (n = 16/4.16%), and malignant neoplasms (n = 342/88.83%). Supplementary Table 2: estimates of life survival and risk functions in 95 cases with avail [file 6890707.f1.zip › 6890707.f1/Supplementary -Figure 2C.jpeg]

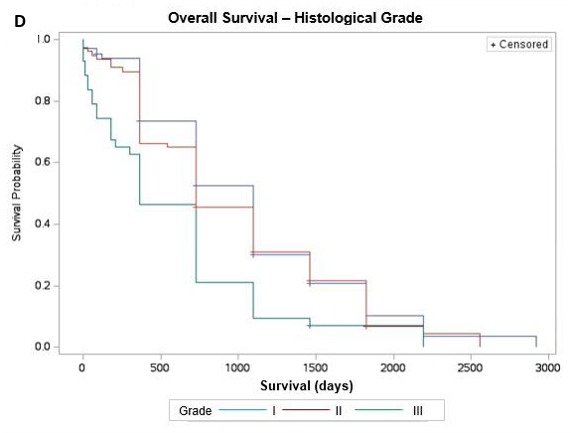

Supplement: Supplementary Materials — Supplementary Figure 1: cancer-specific survival and disease-free interval Kaplan–Meier curve of female dogs with malignant mammary neoplasms. Malignant mammary neoplasms are classified according to (A) CSS age: ≤9 years (median: 1.460 days) and >9 years (median: 365 days); (B) DFI age: ≤9 years (median: 1.460 days) and >9 years (median: 365 days); (C) DFI pseudocyesis: present (median: 1.825 days) and absent (median: 730 days); (D) CSS disease history: negative (median: 730 days) and positive (median: 547 days); (E) CSS clinical staging: initial (median: 730 days) and advanced (365 days); (F) DFI clinical staging: initial (median: 730 days) and advanced (365 days); (G) CSS histological grade: I (median: 1.095 days), II (1.095 days), and III (365 days), respectively; (H) DFI histological grade: I (median: 1.095 days), II (1.095 days), and III (180 days), respectively; (I) CSS ulceration: present (median: 365 days) and absent (median: 730 days); (J) DFI ulceration: present (median: 365 days) and absent (median: 730 days). Supplementary Figure 2: overall survival Kaplan–Meier curve of female dogs with malignant mammary neoplasms. Malignant mammary neoplasms are classified according to (A) age: ≤9 years (median: 1.095 days) and >9 years (median: 365 days); (B) pseudocyesis: present (median: 1.095 days) and absent (median: 730 days); (C) clinical staging: initial (median: 730 days) and advanced (365 days); (D) histological grade: I (median: 1.095 days), II (730 days), and III (365 days), respectively; (E) ulceration: present (median: 730 days) and absent (median: 730 days). Supplementary Table 1: histopathological classification and frequency in % of 385 cases of mammary tumors diagnosed in female dogs treated at the UFV Veterinary Hospital, which were classified into non-neoplastic lesions (n = 27/7.01%), benign neoplasms (n = 16/4.16%), and malignant neoplasms (n = 342/88.83%). Supplementary Table 2: estimates of life survival and risk functions in 95 cases with avail [file 6890707.f1.zip › 6890707.f1/Supplementary -Figure 2D.jpeg]

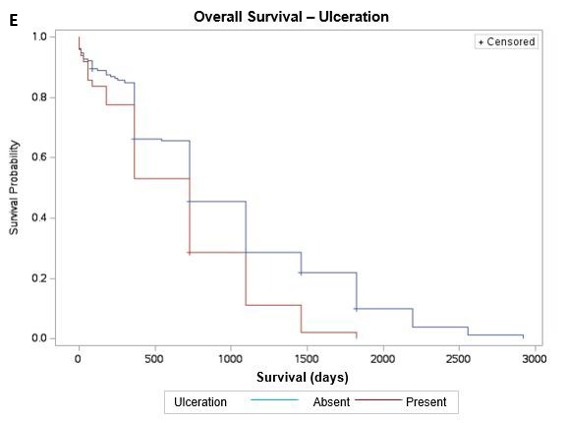

Supplement: Supplementary Materials — Supplementary Figure 1: cancer-specific survival and disease-free interval Kaplan–Meier curve of female dogs with malignant mammary neoplasms. Malignant mammary neoplasms are classified according to (A) CSS age: ≤9 years (median: 1.460 days) and >9 years (median: 365 days); (B) DFI age: ≤9 years (median: 1.460 days) and >9 years (median: 365 days); (C) DFI pseudocyesis: present (median: 1.825 days) and absent (median: 730 days); (D) CSS disease history: negative (median: 730 days) and positive (median: 547 days); (E) CSS clinical staging: initial (median: 730 days) and advanced (365 days); (F) DFI clinical staging: initial (median: 730 days) and advanced (365 days); (G) CSS histological grade: I (median: 1.095 days), II (1.095 days), and III (365 days), respectively; (H) DFI histological grade: I (median: 1.095 days), II (1.095 days), and III (180 days), respectively; (I) CSS ulceration: present (median: 365 days) and absent (median: 730 days); (J) DFI ulceration: present (median: 365 days) and absent (median: 730 days). Supplementary Figure 2: overall survival Kaplan–Meier curve of female dogs with malignant mammary neoplasms. Malignant mammary neoplasms are classified according to (A) age: ≤9 years (median: 1.095 days) and >9 years (median: 365 days); (B) pseudocyesis: present (median: 1.095 days) and absent (median: 730 days); (C) clinical staging: initial (median: 730 days) and advanced (365 days); (D) histological grade: I (median: 1.095 days), II (730 days), and III (365 days), respectively; (E) ulceration: present (median: 730 days) and absent (median: 730 days). Supplementary Table 1: histopathological classification and frequency in % of 385 cases of mammary tumors diagnosed in female dogs treated at the UFV Veterinary Hospital, which were classified into non-neoplastic lesions (n = 27/7.01%), benign neoplasms (n = 16/4.16%), and malignant neoplasms (n = 342/88.83%). Supplementary Table 2: estimates of life survival and risk functions in 95 cases with avail [file 6890707.f1.zip › 6890707.f1/Supplementary -Figure 2E.jpeg]
